# PlGF Repairs Myocardial Ischemia through Mechanisms of Angiogenesis, Cardioprotection and Recruitment of Myo-Angiogenic Competent Marrow Progenitors

**DOI:** 10.1371/journal.pone.0024872

**Published:** 2011-09-28

**Authors:** Hiroto Iwasaki, Atsuhiko Kawamoto, Marc Tjwa, Miki Horii, Saeko Hayashi, Akira Oyamada, Tomoyuki Matsumoto, Shigefumi Suehiro, Peter Carmeliet, Takayuki Asahara

**Affiliations:** 1 Stem Cell Translational Research, Institute of Biomedical Research and Innovation/RIKEN Center for Developmental Biology, Kobe, Japan; 2 Department of Cardiovascular Surgery, Osaka City University Graduate School of Medicine, Osaka, Japan; 3 The Center for Transgene Technology and Gene Therapy, K.U.Leuven, Leuven, Belgium; 4 Department of Transgene Technology and Gene Therapy, VIB, Leuven, Belgium; 5 Department of Regenerative Medicine Science, Tokai University School of Medicine, Isehara, Japan; Leiden University Medical Center, The Netherlands

## Abstract

**Rationale:**

Despite preclinical success in regenerating and revascularizing the infarcted heart using angiogenic growth factors or bone marrow (BM) cells, recent clinical trials have revealed less benefit from these therapies than expected.

**Objective:**

We explored the therapeutic potential of myocardial gene therapy of placental growth factor (PlGF), a VEGF-related angiogenic growth factor, with progenitor-mobilizing activity.

**Methods and Results:**

Myocardial PlGF gene therapy improves cardiac performance after myocardial infarction, by inducing cardiac repair and reparative myoangiogenesis, via upregulation of paracrine anti-apoptotic and angiogenic factors. In addition, PlGF therapy stimulated Sca-1^+^/Lin^−^ (SL) BM progenitor proliferation, enhanced their mobilization into peripheral blood, and promoted their recruitment into the peri-infarct borders. Moreover, PlGF enhanced endothelial progenitor colony formation of BM-derived SL cells, and induced a phenotypic switch of BM-SL cells, recruited in the infarct, to the endothelial, smooth muscle and cardiomyocyte lineage.

**Conclusions:**

Such pleiotropic effects of PlGF on cardiac repair and regeneration offer novel opportunities in the treatment of ischemic heart disease.

## Introduction

Human placental growth factor (PlGF) was originally discovered by Persico *et al* in 1991 as an angiogenic factor [1]. Loss or inhibition of PlGF does not affect normal health, but impairs angiogenesis in pathological conditions [2], [3]. Because PlGF has a higher disease-specific activity than VEGF and other cytokines, and does not affect quiescent vessels in healthy tissues, it is an attractive therapeutic target. An increasing number of reports has now documented the role of PlGF in the angiogenic switch in numerous malignant, inflammatory and ischemic disorders [3]–[6]. Interestingly, cardiac PlGF expression levels predict the improvement of left ventricular function in patients with acute MI [7]. Apart from activating downstream signaling through its own receptor VEGFR-1 (also termed Flt-1), PlGF also transactivates VEGFR-2 (also termed Flk-1) via an intermolecular crosstalk between Flt-1 and Flk-1 [8]. Although PlGF and VEGF activate a similar signaling pathway, the bioactivity of PlGF for angiogenesis is not completely the same as that of VEGF. A recent investigation revealed that anti-PlGF therapy inhibits the growth of VEGFR-inhibitor resistant tumors without affecting healthy vessels [9]. This important finding indicates that PlGF might also have unique potency that is distinct from VEGF in not only tumor angiogenesis but also ischemic neovascularization. Besides affecting endothelial cells (ECs) and smooth muscle cells (SMCs), PlGF is also a potent BM progenitor-active cytokine. Transplantation of BM from wild-type donors into PlGF knockout recipients rescues the angiogenic defects [2], while PlGF promotes the mobilization of marrow-derived hematopoietic and endothelial progenitors [10], [11].

Infusion of PlGF protein or adenoviral PlGF gene transfer enlarges collateral branches and angiogenesis in skin and ischemic limbs in preclinical animal models [3], [12]. Systemic delivery of recombinant PlGF also stimulates neovascularization in the infarct scar in mice when given during a one week period after MI.^3^ A recent study revealed that intramyocardial administration of PlGF1 protein increases endothelial cell density in the rat infarcted myocardium, inhibits left ventricular (LV) dilatation and preserves LV global function [13]. However, the effect of PlGF on LV infarct size and cardiac regeneration via the recruitment of BM progenitors has not been studied previously. In addition, the therapeutic consequences of PlGF delivery on long-term cardiac performance after the arrest of PlGF administration remain unknown. This is relevant, as various angiogenic factors induce only a transient angiogenic response as long as they are being administered, and the newly formed vessels often regress once angiogenic therapy is arrested [14]. Since injection of naked DNA is generally considered to be safer than viral gene transfer, we assessed the therapeutic potential of local intramyocardial delivery of a PlGF expression DNA vector.

Our findings indicate that PlGF improved cardiac performance after acute MI by inducing not only local angiogenesis, but also vasculogenesis and cardiomyogenesis via recruitment of BM-derived progenitors to the infarcted myocardium. The present data offer novel therapeutic opportunities for PlGF gene therapy in patients with ischemic heart disease.

## Materials and Methods

### Experimental animals

Male Sprague-Dawley Rats (Charles River Laboratories, Tokyo, Japan) aged 6 to 7 weeks and weighing 160∼185 g were used for functional assessment of the present study. Male C57BL/6 mice (Charles River Laboratories) aged 7 to 9 weeks were used for proliferation and migration assay in BM cells. GFP transgenic mice (GFP-Tg mice; C57BL/6TgN [act EGFP] Osb Y01) were used in the bone marrow transplantation (BMT) study as donor mice, whereas female athymic nude rats (F344/N Jcl rnu/rnu) (CLEA Japan, Tokyo, Japan) aged 4 weeks and weighing 58∼70 g were used for the BMT study as recipients. All experimental procedures were conducted in accordance with the Japanese Physiological Society Guidelines for the Care and Use of Laboratory Animals and the study protocol was approved by the Ethics Committee in RIKEN Center for Developmental Biology (H17-483). All imaging experiments of infarcted rat hearts were performed in conformance with the SPring-8 Guideline for Care and Use of Laboratory Animals in synchrotron radiation microangiography (SRM) examination (BL28B2).

### Construction of human PlGF plasmid and transfection in vitro

TA cloning was performed according to the manufacturer's instructions (Invitrogen, Carlsbad, CA) [15]. The specific primer for 5′-based on the sequence of human placental growth factor 1 (PlGF 1) cDNA was 5′-TTA AAG CTT CCC TCG GGA CGT-3′ and 5′-TAA ATA CTC GAG CCG GGT GCG-3′. PCR products were ligated into PCR-II TOPO vector (Invitrogen). PlGF cDNA was released as *Hind III* and *XhoI* fragment and subcloned into pcDNA3 plasmid (Invitrogen). Then, ires EGFP (ires: internal ribosomal entry site) was subcloned between the *XhoI* and *XbaI* sites. In brief, the expression vector pcDNA3 consisted of a 5.4 Kb plasmid that includes the human PlGF1 coding gene, transcriptionally regulated by the cytomegalovirus promoter/enhancer, referred to as plasmid of human PlGF1 (pPlGF1). An SV40 terminator is located 3′ to the PlGF1 coding gene or ires-EGFP. Preparation, purification and quality control analyses of the plasmid from transformed E. coli cultures were performed under GMP conditions. The purified plasmid was stored in vials at −80°C. A total of 100, 500 or 1,000 µg of DNA in 5 aliquots of 150 µl of sterile saline was prepared for *in vivo* study. To confirm the expression and secretion of PlGF protein following pPlGF1 transfer *in vitro*, RT-PCR and western blotting using transfected H9C2 cells (rat cardiomyoblast cell line) 2 days after transfection was performed according to the manufacturer's instructions (Roche, Penzberg, Germany). Plasmid encoding IRESeGFP and empty vector (pcDNA3) were also used as a negative control. In brief, these plasmids were transiently transfected into H9C2 cells (1×10^5^ cell/well) using FUGENE 6 transfection reagent (Roche), and cells were allowed to recover for 24 hours in Dulbecco's Modified Eagle Medium (DMEM) (Gibco BRL, Grand Island, NY) containing 10% fetal bovine serum (FBS) (Hyclone Laboratories, Logan, UT). The following day, cells were washed and incubated for 48 hours in DMEM with 10% FBS.

### Induction of myocardial infarction and gene transfer

Six-week-old male Sprague-Dawley Rats were anesthetized with ketamine and xylazine (60 mg/kg and 10 mg/kg, respectively, IP). Myocardial infarction (MI) was induced by ligating LAD as described previously. The proximal left coronary artery under the tip of the left atrial appendage is ligated using a 6-0 prolene suture. Septal branches are not ligated. Proximal LAD ligation in a rat model creates a reproducibly large lateral wall infarction. Twenty minutes after MI, rats received intramyocardial gene transfer of 500 µg empty vector (Mock) or 500 µg pPlGF1 resuspended with 80 µl of PBS or the same volume of PBS without plasmid (n = 8 in each group). After gene transfer was completed, the thorax was closed.

### Echocardiography and hemodynamic measurements

Transthoracic echocardiography (SONOS 5500, Philips Medical Systems) was performed to evaluate LV function immediately before and 5 and 28 days after MI, as described previously. Under general anesthesia with ketamine and xylazine, LV end-diastolic diameter (LVEDD), LV end-systolic diameter (LVESD) and fractional shortening (FS) were measured at the midpapillary muscle level. Regional wall motion score (RWMS) was evaluated per published criteria. Immediately after the final echocardiography on day 28, the rats underwent cardiac catheterization for more invasive and precise assessment of global LV function as described previously. A 2.0 Fr micromanometer-tipped conductance catheter (SPR 838, Millar Instruments Inc., Houston, TX) was inserted via the right carotid artery into the LV cavity. LV pressure and its derivative (dP/dt) were continuously monitored using a multi-channel recording system (Pressure-Volume Conductance System ARIA and Pressure-Volume Analysis Using P-V Analysis Software [Millar Instruments Inc.] and PowerLab® DAQ System [ADInstruments, Australia]). HR, EF, +dP/dt and −dP/dt were continuously recorded for 20 minutes. All data were acquired under stable hemodynamic conditions. All procedures and analyses were performed by an experienced researcher who was blinded to the treatment. The authors randomly assigned the animals to each treatment group.

### Tissue harvesting

All rats were killed 4, 7 and 28 days after gene transfer with an overdose of ketamine and xylazine. At necropsy, hearts were sliced in a broad-leaf fashion into 4 transverse sections from apex to base, embedded in OCT compound, snap-frozen in liquid nitrogen, and stored at −80°C for Masson-trichrome staining and immunohistochemistry. Rat hearts in OCT blocks were sectioned, and 5 µm serial sections were collected on slides, followed by fixation with 4.0% paraformaldehyde at 4°C for 5 minutes and stained immediately. Total RNA for RT-PCR and real-time PCR, and protein for western blotting was isolated by selective dissection of the fibrosis, peri-infarct and remote areas in the LV myocardium.

### Morphometric evaluation of capillary density and infarct size

Histochemical staining with isolectin B4 (Vector Laboratories, Burlingame, CA) was performed, and capillaries were recognized as tubular structures positive for isolectin B4. Histological capillary density was evaluated by morphometric examination of 5 randomly selected fields of tissue sections recovered from segments of LV myocardium subserved by the occluded LAD. To elucidate the severity of myocardial fibrosis, Masson-trichrome staining was performed on frozen sections from each tissue block, and the stained sections were used to measure the average ratio of fibrosis area to the entire LV area (% fibrosis area). All morphometric studies were performed by 2 examiners who were blinded to treatment.

### RT-PCR analysis of ischemic heart tissue

Total RNA was obtained from tissues of rat heart (fibrosis, peri-infarct and remote areas) at days 3, 7 and 14 using Tri-zol (Life Technologies, Grand Island, NY) according to the manufacturer's instructions. In brief, the first-strand cDNA was synthesized using the RNA LA PCR Kit Ver1.1 (Takara, Otsu, Japan), amplified by Taq DNA polymerase (Advantage–GC cDNA PCR Kit (Clontech, Mountain View, CA) and AmpliTaq Gold DNA polymerase (Applied Biosystems, Foster City, CA)). PCR was performed using a PCR thermalcycler (MJ Research PTC-225, Bio-Rad Laboratories, Waltham, MA). The total (human and rat) β-actin was amplified by Taq DNA polymerase (AmpliTaq Gold DNA polymerase, Applied Biosystems) using the following conditions: 35 cycles of 30 seconds initial denaturation at 94°C, annealing at 56°C for 1 minute, and 30 seconds of extension at 72°C according to the manufacturer's instructions. Human PlGF (hPlGF) was amplified by Taq DNA polymerase (Advantage–GC cDNA PCR Kit) under the following conditions: 37 cycles of 30 seconds initial denaturation at 94°C, annealing at 68°C for 3 minute, and 7 minutes of elongation at 64°C according to manufacturer's instructions. Subsequently, PCR products were visualized in 1.5% ethidium bromide-stained agarose gels. Human heart RNA distributed from Clontech (premium RNA) was used as positive control. Primers: To avoid interspecies cross-reactivity of the primer pairs between human and rat genes, we designed the following human-specific primers using Oligo software (Takara). None of the primer pairs showed cross-reactivity to rat genes (data not shown). PlGF primer sequence (224 bp): sense TTA GGA GCA GCA TGG TGA CAT TGG C; antisense CGG CTC GTC AGA GGT GGA AGT GGT A; β-actin primer sequence (427 bp): sense ACC CTA AGG CCA ACC GTG AAA; antisense TCA TTG CCG ATA GTG ATG ACC TGA C.

### Real-time PCR analysis of gene expression

Real-time PCR was performed on an ABI PRISM 7000 sequence detector (Applied Biosystems) as described previously. Total RNA was prepared as described above. The expression of related genes was quantified using the SYBR green reagent (2× SYBR Green Supermix; Bio-Rad, Hercules, CA) following the manufacturer's instructions on a Bio-Rad Cycler. Multiple PCR was performed under optimized conditions: 95°C denatured for 3 minutes, followed by 40 cycles of 45 seconds at 94°C, 45 seconds at 55°C, and 45 seconds at 72°C using the primers as described below. Primer for human-specific PlGF was confirmed not to cross-react with rat genes by using rat heart samples. Similarly, specificity of mouse-specific primers was demonstrated using rat tissue samples (data not shown).

Primers: hPlGF, forward, 5–TGG AGC ACA TGT TCA GCC C-3, reverse, 5-GCT GCA TGG TGA CAT TGG C-3; rPlGF, forward, 5-GTT GGC TGT GCA CTC CCA G-3, reverse, 5–GTT GGC TGT GCA CTC CCA G-3; rFlt-1, forward, 5–TGT CGG CTG CAG TGT GTA AGT-3, reverse, 5–CCC AAT TGC TGG ATA TCT GGA; rVEGF, forward, 5–GAC AGA AGG GGA GCA GAA AG-3, reverse, 5–AAG CAA TGC TGT TGT CAC CTT CCC-3; rSDF-1, forward, 5–CGT GGG CTC TGA GTT TTC GA-3, reverse, 5–GAT TGA AAC CCA GAA TCC CC- 3; rAng-1, forward, 5 –CGA ATT CAA CAT GGG CAA TG- 3, reverse, 5 –TCG CAC TCT CAC GGC AGT T-3; rAng-2, forward, 5 –CTA CAG GAT TCA CCT TAC AGG ACT CA-3, reverse, 5 –CTT CCT GGT TGG CTG ATG CT-3; rIGF-1, forward, 5 –GTC CGC TGC AAG CCT ACA AA-3, reverse, 5 –TGA GTC TTG GGC ATG TCA GTC T-3; rIGFR, forward, 5 –CAC ACC GGA CGA CAA CAC A, reverse, 5 – CAC GCA CAC GCC TTT GTA GT -3; mFlt-1, forward, 5–TCG GCC ATC ATC TGA ATG TG-3, reverse, 5 –TCC ACG ATC ACC ATG AGA-3; mFlk-1, forward, 5–GAG CTC TCC GTG GAT CTG AA-3, reverse, 5–AAC AAG CCT GAG CTG GC-3; mCXCR4, forward, 5–GAA TGA GCG TCT AGG CAG GA-3, reverse, 5–CAG CTG AGG ATC ACG GCT-3; and β-actin, forward, 5 – AAG TCC CTC ACC CTC CCA AAA G-3, reverse, 5 –AAG CAA TGC TGT CAC CTT CCC- 3. No other products were amplified because melting curves showed only one peak in each primer pair. Fluorescence signals were measured over 40 PCR cycles. The cycle number at which the signals crossed a threshold set within the logarithmic phase was recorded. For quantitation, we evaluated the difference in cycle threshold between the AP-treated group and vehicle control of each gene. The efficiency of amplification of each pair of primers was determined by serial dilutions of templates and all were 0.9. Each sample was normalized with the loading reference β-actin. The *Ct* values used were the means of triplicate replicates. Experiments were repeated at least three times.

### ELISA assay

Total hPlGF and rVEGF protein concentrations were determined by a solid-state ELISA system with Quantikine PlGF and VEGF ELISA kit (R&D Systems) according to the manufacturer's instructions. Each plasma sample was tested in triplicate.

### TUNEL assay

We obtained rat heart samples 4 days after gene transfer. Frozen heart specimens were sectioned as described above. Apoptosis was detected by the TUNEL assay (Roche) according to the manufacturer's instruction. Immunohistochemistry with cTn-I was performed to identify mature cardiomyocytes (CMCs) in rat hearts. The number of apoptotic CMCs was evaluated by counting the number of double-positive cells for TUNEL and cTn-I in 5 randomly selected fields within the rat ischemic myocardium at day 4. The % apoptotic CMCs was calculated as the ratio of the number of apoptotic CMCs to the total number of CMCs. The morphometric examination was performed by two investigators blinded to the treatment group.

### Western blotting

Western blot analysis was performed as described previously. Heart tissue and BM- Sca-1^+^/Lin^−^ (SL) cell extracts were subjected to SDS-PAGE, transferred to PVDF membranes and, following antibody incubations, developed with enhanced chemiluminiscence detection kit. Loading controls were performed with the following antibodies. In brief, LV specimens (muscle) obtained for Western blotting were snap-frozen in liquid nitrogen without OCT compound. Specimens and cells were sheared with a 23G needle after homogenization in 10 volumes of T-PER® lysis buffer (Tissue Protein Extraction Reagent) (PIERCE, Rockfold, IL) with protease inhibitor (Roche). After normalizing for protein content, 50 µg of each sample was electrophoresed on a 12.0% SDS-polyacrylamide gel following 5 minutes of denaturation at 95°C. A wet transfer apparatus was used to transfer proteins to PVDF membranes, which were then blocked with 5% nonfat dry milk and FBS in PBS containing 0.05% Tween 20. Immunoblotting was performed using the monoclonal antibody against PlGF (1∶500 dilution) and polyclonal antibodies against α-tubulin (1∶3000) (both from Abcam, Cambridge, UK), Bcl-xL (1∶500) (Southern Biotech, Birmingham, AL) and Akt (1∶1000) (Cell Signaling Beverly, MA). The secondary antibodies for immunoblotting are as follows: anti-rabbit IgG HRP for PlGF, α-tubulin and Akt, and anti-mouse IgG HRP for Bcl-X_L_ (both from Amersham Bioscience, Buckinghamshire, UK). PI3K-Akt pathway was detected by western blotting using PathScan Multiplex Western Mixture I (Cell Signaling) according to the manufacturer's instructions.

### Growth factors

Recombinant human growth factors and cytokines, i.e. stem cell factor (rh-SCF) and thrombopoietin (rh-TPO) were kindly donated by Kirin Brewery Co (Tokyo, Japan). rh-Flt-3 ligand and insulin-like growth factor-1 (rh-IGF-1) were purchased from Peprotec and heparin from Shimizu Pharmaceutical Co, Ltd., (Tokyo, Japan).

### Isolation of SL cells

BM-MNCs were isolated from the BM of C56BL/6 mice or xeno-rats. The Sca-1^+^ and lineage^−^ (Sca-1^+^/lin^−^) fraction of the BM- mononuclear cell (MNC) population was sorted by fluorescence-activated cell sorting (FACSAria™) (Becton Dickinson Immunocytometry Systems, Mountain View, CA). Prior to the sorting procedure, BM-MNCs were stained with a cocktail of biotinylated monoclonal antibodies against various lineage markers, including B220/CD45R (clone RA3-6B2); CD11b/Mac-1 (clone Mi/70); Gr-1 (clone RB6-8C5); Thy1.2 (clone 53-2.1); CD3e (clone 145-2C11); CD4 (clone RB6-8C5); CD8 (clone 53-6.72); and TER 119 (clone Ly-76; all from BD Pharmingen, San Diego, CA) for 20 minutes at 4°C. The cells were stained with a PE-conjugated anti-Sca-1 antibody (BD Pharmingen) and APC-conjugated streptavidin (BD pharmingen) for 20 minutes at 4°C.

### Expansion culture of isolated SL cells (proliferation assay)

Isolated 1.5×10^3^ BM-SL cells were cultured in each well of a 96-well tissue culture dish (Primaria™, BD Falcon, MA) using serum free culture media (STEM SPAN™, Stem Cell Tec, Tokyo, Japan) containing 100 ng/ml hSCF, 100 ng/ml hFlt-3 ligand, 20 ng/ml hTPO in the presence or absence of 50 ng/ml PlGF for 7 days. To assess the effect of PlGF on the proliferation activity of SL cells, the total number of expanded SL cells in each well was counted in a blinded manner under ×200 magnification.

### BrdU incorporation

Cell proliferation was also evaluated by measuring the incorporation of BrdU, indicating the S phase of the cell cycle. BrdU incorporation was detected by using kits purchased from Roche according to the manufacturer's instructions. For immunofluorescent assay, cells were cultured in the presence or absence of PlGF (0, 0.1, 1 or 50 ng/ml) for 4 days. BrdU was added to the cells and reincubated for 4 hours. After removing the culture medium, the cells were fixed and DNA was denatured in one step by adding FixDenat. Thereafter, the anti-BrdU-POD, which binds to the incorporated BrdU in newly synthesized cellular DNA, was added for 90 minutes. Further development was accomplished with substrate solution for 3 minutes. Light absorbance at 490 nm in each plate was quantified by ELISA plate reader (Bionetics Laboratory) [8]. The absorbance value for each concentration of PlGF was corrected by subtracting the value of the blank (without BrdU loading).

### Migration assay

To investigate PB-SL cell migration activity, a modified Boyden chamber assay was performed using a 48-well microchemotaxis chamber (NeuroProbe). PlGF was diluted to the appropriate concentration in DMEM, supplemented with 0.1% BSA, and 30 µl of the final dilution was placed in the lower compartment of a Boyden chamber (PlGF concentration: 1, 20 or 100 ng/ml). Isolated PB-SL cells (1×10^4^ cells) were suspended in 50 µl of DMEM supplemented with 0.1% BSA, and antibiotics for 6 hours at 37°C. The filter was then removed, and the cells on the filter were counted manually in random high-power fields (×100) in each well. All groups were studied at least in triplicate.

### Experimental animals in BMT

To evaluate the effect of pPlGF1 on BM-derived stem/progenitor cells, we established BMT from GFP transgenic mice to nude rats (xeno-rats). Low-density BM-MNCs of GFP transgenic mice were isolated by density-gradient centrifugation with Histopaque-1083 (Sigma). The immunodeficient rats were lethally irradiated with 8.0 Gy and the 2×10^7^ donor BM-MNCs suspended in 100 µl of PBS were injected intravenously from the tail vein of immunodeficient rats. Four weeks post-BMT, by which time the BM of the recipient rat was reconstituted by donor mice's cells, these xeno-rats underwent induction of MI, and were randomly assigned to treatment with PlGF plasmid, Mock or PBS. The rats were killed 1, 7 and 28 days after treatment.

### EPC colony forming assay (EPC-CFA)

To further investigate the effect of pPlGF1 transfer on the vasculogenic potential of BM-SL cells isolated from xeno-rats, we performed the endothelial progenitor cell (EPC)-CFA using the cells obtained 7 days after the gene therapy, as described below. Isolated SL cells were seeded into each semi-solid culture system. Semi-solid culture medium for EPC-CFA was adjusted using serum free 1.0% methylcellulose in Iscove Modified Dalbecco's Medium (IMDM) (MethoCult™ SF^BIT^ H4236, Stem Cell Tech.), containing 30% FBS (JRH Bioscience), 50 ng/ml rh-VEGF, 50 ng/ml rh-bFGF, 100 ng/ml rh-SCF, 50 ng/ml rh-IGF-1, 20 ng/ml rh-IL3, 50 ng/ml rh-EGF, and 2 IU/ml heparin. Isolated SL cells (500 cells per dish) were seeded into a 35 mm hydrophilic tissue culture dish (Primaria™, BD Falcon) to promote cell adhesion.

In an EPC-CFA, two types of cell clusters, i.e. non-adherent or poorly-adherent cells exhibiting large (20 to 50 µm) and small (10 to 20 µm) cell diameters, respectively usually appear 8 to 12 days after initiation of the assay. At day 10 to 14, the large cells start to adhere to the tissue culture dish and acquire a more spindle-like shape. The small cells gradually start to proliferate without, however, adhering or changing their shape. After 14 to 18 days, these two types of cells were independently recognized as definite colony forming units (CFUs). The CFUs containing large cells (large EPC colony) tightly adhered as sheets of spindle-like cells (50 to 200 µm). In contrast, CFUs containing small cells (small EPC colony) were weakly adherent small round cells. We previously reported that both EPC colonies show acetyl-LDL uptake and are positive for isolectin B4, an EC-specific marker. Both types of colonies also express CD31, VEGFR-2 and VE-cadherin. EPCs in small colonies are more immature and capable of differentiating into more mature EPCs in large EPC colonies, as observed with time-lapse microscopic examination. Eighteen to twenty-one days later, the methylcellulose was resolved and gently removed with a pippetor after adding 1 ml of ice-cold PBS. Then, immediately after adding 1 ml of 5% FCS-EBM-2 medium with endothelial growth supplement kit (EGM-2 MV single quots, Clonetics) without hydrocortisone, two types of adherent colonies, small EPC colony and large EPC colony, were counted under light microscopy.

### Immunofluorescent staining

To detect incorporation of BM-derived SL cells in ischemic myocardium of xeno-rats, double immunohistochemistry was performed with the following antibodies: GFP (Molecular Probes, Carlsbad, CA) and Sca-1 (BD Pharmingen, San Diego, CA). Double staining for GATA4 or MEF2C (Santa Cruz, CA) and GFP or Sca-1 was performed to detect BM-derived cells expressing early cardiomyogenic transcription factor in ischemic myocardium of xeno-rats at day 7. Double staining for GATA6 or Ets-1 (Santa Cruz, CA) and GFP or Sca-1was performed to detect BM-derived cells expressing early vasculogenic transcription factor in the ischemic myocardium of xeno-rats at day 7. Moreover, double staining for cTn-I (Chemicon International) as mature CMC marker and GFP was performed to detect cardiomyogenic plasticity of incorporated BM-derived GFP+ cells at day 28. Double immunohistochemistry with GFP and SMA was performed to detect double-positive cells as BM-derived smooth muscle cells (SMCs) in rat myocardium. Similarly, double immunohistochemistry with isolectin B4 (Vector Laboratories) and GFP was performed to detect double positive cells as BM-derived endothelial cells (ECs) in ischemic myocardium. Immunohistochemistry for SDF-1 (R&D System) was performed to assess the underlying mechanism of enhancement of the recruitment of BM-derived progenitor cells after pPlGF1 gene transfer. The secondary antibodies for each immunostaining were as follows: Alexa Fluor 488 or 594-conjugated goat anti-mouse IgG_2a_ and goat anti-rabbit IgG (Molecular Probes, Carlsbad, CA) for GFP staining, Alexa Fluor 594-conjugated goat anti-mouse IgG_1_ (Molecular Probes) for Sca-1 and SDF-1 staining, Alexa Fluor 488 or 594-conjugated goat anti-rabbit IgG (H+L) (Molecular Probes) for GATA4, GATA6 or Ets-1 staining, Alexa Fluor 488 or 594-conjugated mouse anti-goat IgG (Molecular Probes) for MEF2C staining, Alexa Fluor 488-conjugated goat anti-mouse IgG_2a_ (Molecular Probes) for cTn-I staining and Alexa Fluor 488-conjugated goat anti-mouse IgG_2a_ (Molecular Probes) for SMA. DAPI solution was applied for 5 minutes for nuclear staining.

To detect the growing endothelial cells or cardiomyocytes, sections were immunostained with anti-phospho-Akt (Ser473) rabbit monoclonal antibody (Cell Signaling Technology Inc., Danvers, MA) or anti-ACTIVE® MAPK rabbit polyclonal antibody (pTEpY) (Promega, Madison, WI) and Alexa Fluor 594-conjugated isolectin B4 (Invitrogen, Frederick, MD) or mouse anti-cardiac troponin I antibody (Millipore, Billerica, MA) according to the manufacturer's instructions, followed by the incubation with the secondary antibody Alexa Fluor 488-conjugated donkey anti-rabbit IgG or Alexa Fluor 594-conjugated donkey anti-mouse IgG2b (Invitrogen). The sections were counterstained with DAPI. Images were captured with a BX51 light microscope (Olympus, Tokyo, Japan).

To detect the integration of the BM-derived cells with host cardiomyocytes in ischemic myocardium of xeno-rats, immunohistochemistry was performed. Frozen sections were fixed in 4% PFA/PBS for 10 minutes at 4°C and blocked with 5% normal goat serum/0.1% TritonX-100 in PBS. Sections were immunostained with anti-green fluorescent protein (GFP) rabbit polyclonal antibody (Thermo Fisher Scientific, Waltham, MA) and purified mouse anti-connexin-43 mouse monoclonal antibody (Pharmingen, San Diego, CA), followed by the secondary antibodies Alexa Fluor 488-conjugated goat anti-rabbit IgG antibody and Alexa Fluor 594-conjugated goat anti-mouse IgG1 antibody (Invitrogen, Frederick, MD). Sections were additionally stained with mouse anti-cardiac troponin I antibody (Millipore, Billerica, MA), followed by aminomethylcoumarin-conjugated goat anti-mouse IgG(H+L) antibody (Jackson ImunoResearch Laboratories, West Grove, PA).

### Culture of sorted BM Sca-1^+^/Lin^−^ cells for cardiomyogenic and vasculogenic differentiation and quantitative RT-PCR

BM cells were obtained by flushing the tibias and femurs of C57BL/6 mice. The Sca-1^+^/Lin^−^ fraction of the BM cells was sorted by fluorescence-activated cell sorting (FACSAria™) (BD Biosciences, San Jose, CA). Prior to the sorting procedure, Lin^−^ cells were immunomagnetically isolated by depleting mature hematopoietic cells such as T cells, B cells, monocytes/macrophages, granulocytes and erythrocytes using the biotinylated mouse lineage depletion cocktail (IMag, BD Pharmingen, San Jose, CA), containing CD3e, CD11b/Mac-1, CD45R/B220, Gr-1 and TER119. The cells were then stained with phycoerythrin (PE)-conjugated anti-Sca-1 antibody (BD Pharmingen), biotinylated mouse lineage depletion cocktail and streptavidin-APC/Cy7 (BioLegend, San Diego, CA) for 20 minutes at 4°C.

Sorted BM SL cells were plated into a 48-well plate coated with rat vitronectin (Sigma-Aldrich, St.Louis, MO) at a density of 5×10^5^ cells/well and cultured with 2%FBS/EBM-2 supplemented with 10 nM dexamethasone (Sigma-Aldrich), 0, 1, 10 or 100 ng/mL recombinant human PlGF (R&D Systems, Minneapolis, MN), and with or without 1 ng/mL mouse VEGF (PeproTech, Rocky Hill, NJ) for 7 days. Total RNA was then obtained using RNeasy Mini kit (QIAGEN, Hilden, Germany) according to the manufacturer's instructions.

The first-strand cDNA was synthesized using the PrimeScript RT reagent Kit (Takara Bio, Otsu, Japan) and real-time quantitative RT-PCR was performed with ABI Prism 7700 (Applied Biosystems, Foster City, CA) using SYBR Green Master Mix Reagent (Applied Biosystems).

The sequences of primers were as follows:

GATA4

Fw: TCCAGTGCTGTCTGCTCTGAA

Rv: TGGCCTGCGATGTCTGAGT

cTnI

Fw: AGATCTCCGCCTCCAGAAAAC

Rv: TCCATCTCCTGCTTCGCAAT

Flt-1

Fw: TCGGCCATCATCTGAATGTG

Rv: TCCACGATCACCATCAGAGG

VE-cad

Fw: TCGGGAGCATGCCAAGTT

Rv: TGGGCACCCCGTTGTC

eNOS

Fw: CCGAAGCAGCACTCTTGCA

Rv: CCGCAATGAGCCCTTTCTT

GAPDH

Fw: TGTGTCCGTCGTGGATCTGA

Rv: ACCACCTTCTTGATGTCATCATACTT

### Statistical analysis

The results were statistically analyzed using a software package (Statview 5.0, Abacus Concepts Inc., Berkeley, CA). All values were expressed as mean±standard error (mean±SE). Comparisons among more than 3 groups were made using one-way analysis of variances (ANOVAs). Post-hoc analysis was performed using Scheffe's PLSD test. Differences of P<0.05 were considered to denote statistical significance.

## Results

### pPlGF1 upregulates angiogenic factors in ischemic myocardium

**Figure 1:**
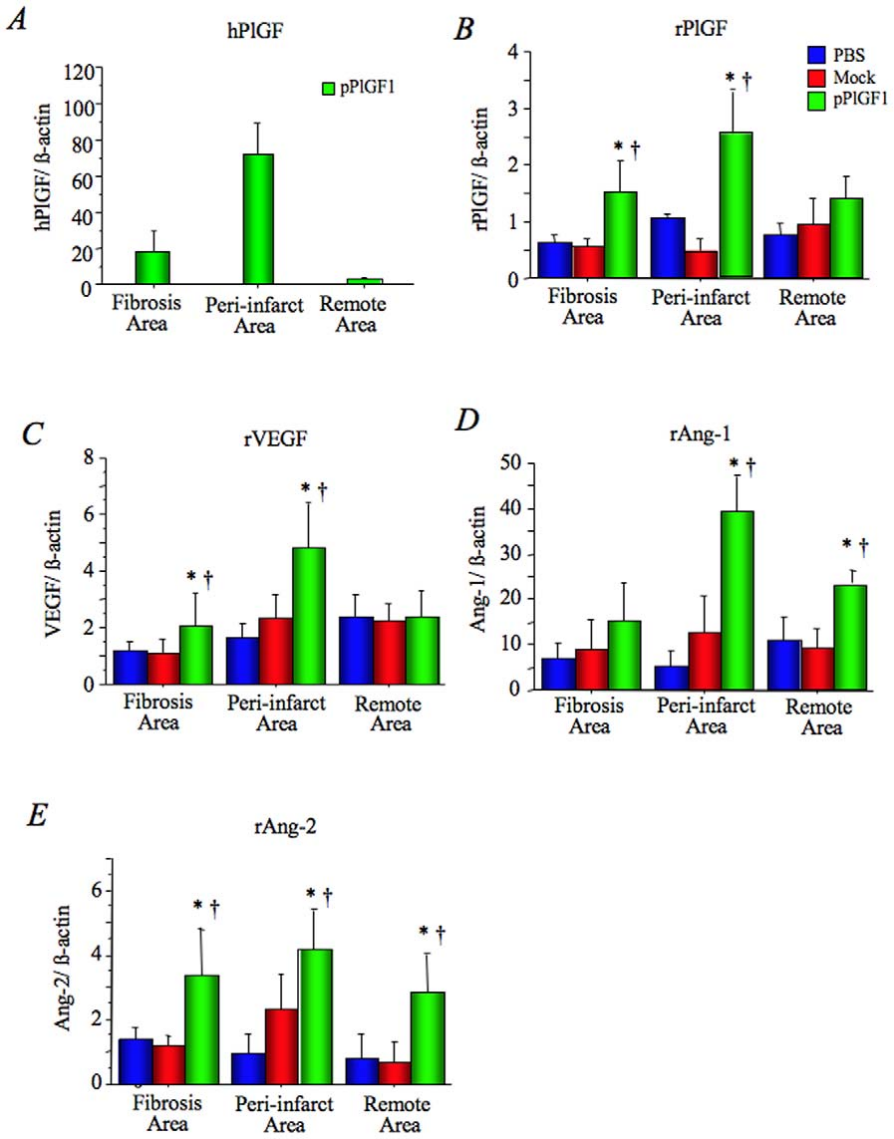
pPlGF1 upregulates angiogenic factors in ischemic myocardium. ***A*** **:** Real-time PCR revealed that gene expression of hPlGF was detectable in fibrosis and peri-infarct areas 4 days post pPlGF1 gene transfer. ***B*** **,** ***C*** **,** Real-time PCR revealed that gene expression of rPlGF and rVEGF in fibrosis and peri-infarct areas was significantly enhanced in the pPlGF1 group compared with controls. ***D*** **,** ***E*** **,** Real-time PCR revealed that expression of rAng-1 in peri-infarct and remote areas and that of rAng-2 in all areas significantly augmented in the pPlGF1 group compared with controls. *, P<0.05 vs PBS; †, P<0.05 vs Mock. (n = 8 in each group).

Using real-time RT-PCR, we also examined whether pPlGF1 gene transfer altered the endogenous myocardial expression of the angiogenic cytokines VEGF (rVEGF), angiopoietin-1 (rAng-1), angiopoietin-2 (rAng-2) as well as of PlGF (rPlGF) at 4 days after the gene transfer. hPlGF transcripts were detectable at day 4 in the peri-infarct borders (**Figure 1** ***A***). The expression of rPlGF and rVEGF in the infarct and peri-infarct borders was upregulated to higher levels in the pPlGF1 group than in the controls (P<0.05) (**Figure 1** ***A, B***). Compared to the control groups, pPlGF1 gene transfer also augmented transcript levels of rAng-1 in the peri-infarct and remote areas, and of rAng-2 in all areas (P<0.05) (**Figure 1** ***C, D***). Thus, pPlGF1 gene transfer transiently elevates the levels not only of hPlGF1, rPlGF, and rVEGF but also of rAng-1 and rAng-2.

### pPlGF1 transfer enhances vascularization and reduces infarct size

**Figure 2:**
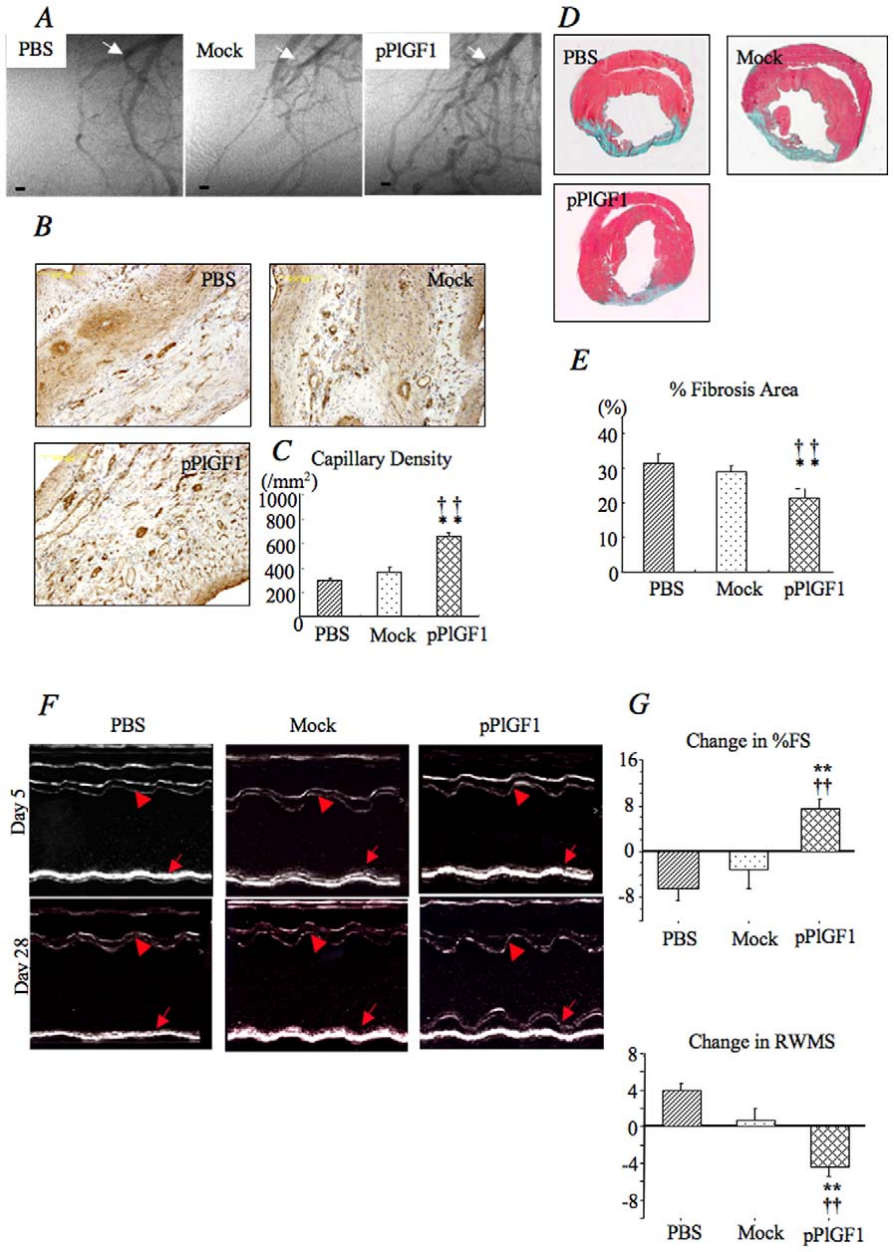
Angiographical or histological evaluation of LV remodeling and myocardial neovascularization after MI. ***A*** **,** Representative microangiographic images 28 days after PBS, Mock or pPlGF1 injection (7.0×7.0 mm; scale bar; 100 µm) (arrow: ligation point). ***B*** **,** Representative histochemical staining for isolectin B4 at day 28 (×20). ***C*** **,** Representative Masson-trichrome staining at day 28. ***D*** **,** Capillary density in rats receiving pPlGF1, Mock or PBS at day 28. **, P<0.01 vs PBS; ††, P<0.01 vs Mock. ***E*** **,** Ratio of fibrosis area/entire LV area (% fibrosis area) at day 28. **, P<0.01 vs PBS; ††, P<0.01 vs Mock. (n = 8 in each group). **LV functional evaluation by echocardiography.** ***F*** **,** Representative recording of M-mode echocardiography 5 and 28 days after pPlGF1, Mock or PBS injection (arrow, endocardium in lateral wall; arrowhead, endocardium in septal wall). ***G*** **,** Changes in echocardiographic parameters between day 5 and day 28 after gene transfer (n = 8 in all groups). FS, fractional shortening; RWMS, regional wall motion score. **, P<0.01 vs PBS; ††, P<0.01 vs Mock. (n = 8 in each group).

We then examined whether PlGF1 gene transfer stimulates revascularization of the ischemic myocardium. To this end, PBS, 500 µg empty vector (Mock) or pPlGF1 was injected intramyocardially in Sprague Dawley rats 20 minutes after ligation of the LAD. At 28 days after gene transfer, we performed a coronary microangiography on *ex vivo* beating hearts, using a third generation synchrotron radiation microangiography (SRM) [16] to visualize coronary vessels as small as 20 µm in diameter. This analysis revealed that pPlGF1 gene transfer stimulated collateral vessel growth, as more conduit vessels extended into the perfusion territory of the occluded LAD in the pPlGF1 group than the controls (**Figure 2** ***A***). In addition, when counting the density of isolectin B4 stained microvessels, we found that pPlGF1 gene transfer stimulated capillary angiogenesis in the ischemic myocardium (P<0.01 for pPlGF1 vs Mock or PBS) (**Figure 2** ***B,D***).

**Figure 3:**
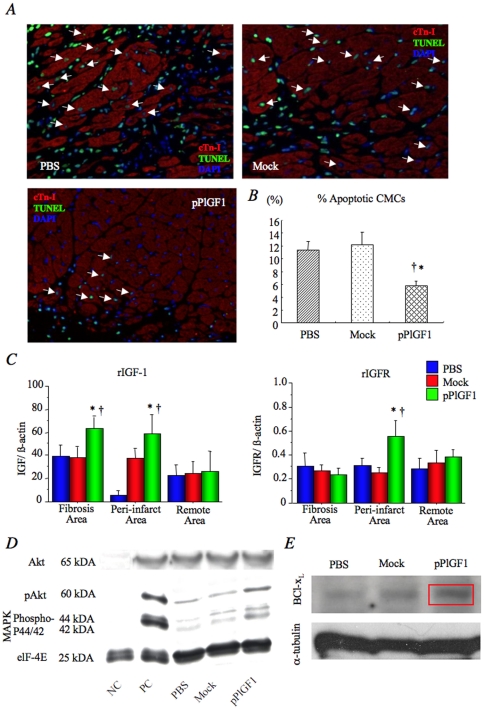
pPlGF1 reduces cardiomyocyte (CMC) apoptosis. ***A*** **:** Cardiac apoptosis was detected by the TdT-mediated dUTP nick end-labeling (TUNEL) assay (green) and cardiac troponin-I (cTn-I) staining. White arrows showed TUNEL-positive CMCs in infarcted myocardium (apoptotic CMCs). ***B*** **:** Bar graph indicates % apoptotic CMCs, which was calculated as the ratio of the number of TUNEL-positive CMCs to the number of total CMCs in the infarcted myocardium. The % apoptotic CMCs on day 4 significantly decreased following pPlGF1 injection compared to Mock and PBS administration. *, P<0.05 vs PBS; †, P<0.05 vs Mock. ***C*** **:** Real-time PCR demonstrated significant upregulation of endogenous IGF-1 in infarcted and peri-infarct areas and IGFR at peri-infarct area after pPlGF1 gene transfer compared with the controls. ***D*** **:** Immunoblotting for phospho-Akt and phospho-p44 or p42 of MAPK revealed enhanced expression of these proteins in the pPlGF1 group than controls. ***E*** **:** Immunoblotting indicated greater expression of Bcl-x_L_ protein in the pPlGF1 group than in the controls. (n = 6 in each group).

Since the infarcted myocardium is well known to develop into a collagen-rich fibrotic scar, we measured the size of the fibrotic area in the LV as an indirect measure of the infarct size. Masson-trichrome staining at 28 days after infarction revealed that the fibrotic area was ∼30% smaller in the pPlGF1 group than the control groups (P<0.01 for pPlGF1 vs Mock and PBS) (**Figure 2** ***C, E***). Since the standardized LAD ligation procedure resulted in a similar ischemic myocardial area at risk in each group, the finding that pPlGF1 gene transfer reduced the infarct size indicates that more CMCs survived the ischemic insult in this group. Indeed, cardiomyocyte apoptosis was significantly more inhibited in the pPlGF1 group than in the controls (P<0.05 for pPlGF1 vs Mock and PBS) (**Figure 3**).

### pPlGF1 improves left ventricular function after acute MI

To determine whether pPlGF1 gene transfer improved cardiac performance, we performed echocardiography at day 5 and 28 after gene transfer. There were no significant differences in preoperative echocardiographic parameters, LVEDD, LVESD, FS and RWMS among the pPlGF1, Mock and PBS groups (not shown). Cardiac performance is known to deteriorate at later stages after MI and is, in fact, becoming an increasingly more frequent cause of cardiac failure in post-MI survivors. Echocardiography revealed comparable functional parameters in all groups at day 5 after gene transfer, but an improvement in cardiac performance selectively in the pPlGF1 group at later times. Indeed, when determining the change in FS between day 5 and 28, the FS was increased after pPlGF1 but reduced in the control groups (**Figure 2** ***F,G***). Similarly, myocardial wall dyskinesis was reduced in the pPlGF1 group but increased in the controls (P<0.01 for pPlGF1 vs Mock and PBS) (**Figure 2** ***F,G***).

### pPlGF1 gene transfer reduces cardiomyocyte apoptosis *in vivo*

The reduced infarct size after pPlGF1 gene transfer might be attributable, at least in part, to the possibility that fewer CMCs died immediately after LAD ligation. As apoptosis is generally regarded as one of the main mechanisms responsible for ongoing myocardial degeneration after acute MI, we analyzed the effect of pPlGF1 transfer on CMC apoptosis by double staining for TdT-mediated dUTP nick end-labeling (TUNEL) and cardiac troponin-I (cTn-I) at 4 days after gene transfer. This analysis revealed that pPlGF1 gene transfer reduced the number of TUNEL^+^ CMCs in the infarcted myocardium (% apoptotic CMCs: pPlGF1, 5.9±1.0%; Mock, 12.1±2.2%; PBS, 11.5±1.7%, P<0.01 for pPlGF1 vs Mock and PBS) (**Figure 3** ***A, B***).

We next sought to determine the molecular mechanism by which pPlGF1 transfer reduced CMC apoptosis. As insulin-like growth factor 1 (IGF-1) and its receptor (IGFR) are well known for their anti-apoptotic effects on CMCs, we measured endogenous transcript levels of rat IGF-1 (rIGF-1) and rat IGFR (rIGFR), by real-time RT-PCR, 4 days after pPlGF1 transfer. Expression of rIGF-1 and rIGFR was higher in the infarcted and peri-infarct areas in the pPlGF1 group than control groups (P<0.05) (**Figure 3** ***C***). Since survival signals such as IGF-1 activate Akt via phosphorylation by phosphatidylinositol 3-kinase (PI3-kinase) and 3-phosphoinositide-dependent kinase (PDK), we determined the levels of phospho-Akt in the ischemic myocardium at day 4 in each group. Immunoblotting revealed that phospho-Akt levels were elevated significantly more in the pPlGF1 group than in the control groups (**Figure 3** ***D***). Because phosphorylation of p42/p44 MAPK is also well known to be cardioprotective by promoting cell survival, we examined the levels of phospho-p42/p44 in the ischemic myocardium at day 4 in each group. Immunoblotting revealed that phospho-p42/p44 levels were significantly higher in the pPlGF1 group than in the controls (**Figure 3** ***D***). To confirm that pPlGF1 gene transfer induced survival pathways, we also analyzed the expression of *Bcl-x_L_*, one of the *Bcl-2* family of proteins known to inhibit CMC apoptosis and to be upregulated by growth factors. Western blotting revealed that Bcl-x_L_ was overexpressed in the ischemic myocardium at day 4 in the pPlGF1 group as compared to the control groups (**Figure 3** ***E***). Thus, the induction, by pPlGF1 gene transfer, of a survival signaling cascade, involving IGF-1, PI3K-Akt and the anti-apoptotic protein Bcl-*x_L_*, likely contributed to the improved survival of ischemic CMCs after LAD ligation.

### PlGF recruits BM-SL cells to the infarct, directly and indirectly via SDF-1

**Figure 4:**
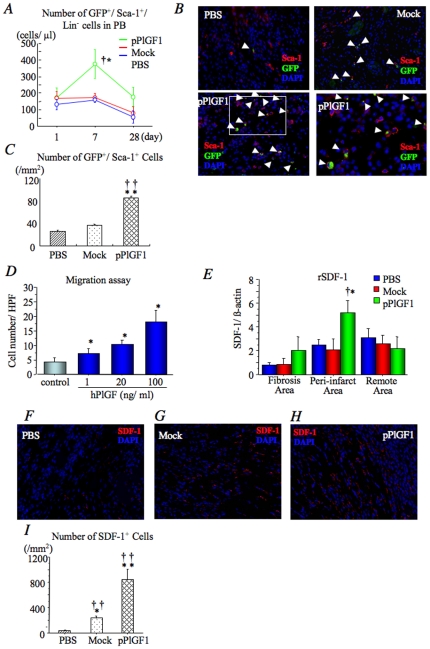
pPlGF1 auguments mobilization of BM progenitors into PB and their recruitment into ischemic myocardium. ***A*** **,** Representative recording of serial FACS analyses of PB GFP^+^/lin^−^ cells isolated from xeno-rats in each group. The number of PB GSL cells 7 days after MI was significantly increased in the pPlGF1 group than controls. *, P<0.05 vs PBS; †, P<0.05 vs Mock. ***B*** **,** Double immunofluorescent staining for Sca-1 and GFP (green) in infarcted myocardium of each group at day 7. Arrows show the double positive cells, which indicate BM-derived immature cells were incorporated into the ischemic area. ***C*** **,** The bar graph showing the number of Sca-1+/GFP+ cells in the ischemic area indicates enhanced recruitment of the BM-derived immature cells by pPlGF1 gene transfer at day 7. **, P<0.01 vs PBS; ††, P<0.01 vs Mock. ***D*** **,** Migratory response of PB-SL cells toward different dosages of PlGF by modified Boyden chamber migration assay. *, P<0.05 vs 0 ng/ml (control). ***E*** **,** Real-time PCR demonstrated that pPlGF1 significantly augmented expression of rSDF-1 mRNA at the peri-infarct area 4 days after MI compared with controls. *, P<0.05 vs PBS; †, P<0.05 vs Mock. ***F–H*** **,** Representative immunofluorescent staining for SDF-1 in PBS (***F***), Mock (***G***) or pPLGF1 (***H***) group 7 days after gene transfer. Red fluorescence indicates SDF-1 protein in cytoplasms. ***I*** **,** Quantification of SDF-1+ cells at peri-infarcted area in rats receiving pPlGF1, Mock or PBS at day7. SDF-1+ cells were significantly increased following pPlGF1 gene transfer. **, P<0.01 vs PBS; *, P<0.05 vs Mock; ††, P<0.01 vs Mock. (n = 8 in each group).

We then assessed whether pPlGF1 gene transfer in bone marrow transplantation/myocardial infarction/gene therapy (BMT/MI/GTx) animals mobilized BM-SL cells from the BM to the infarcted myocardium. FACS analysis at day 7 post-MI revealed a larger number of circulating GSL cells in the peripheral blood (PB) of BMT/MI/GTx animals in the pPlGF1 group than in the control groups (**Figure 3** ***A***). pPlGF1 gene transfer in BMT/MI/GTx animals also mobilized more EPCs from the BM. To identify these BM-derived EPCs in the PB of BMT/MI/GTx animals, real time RT-PCR was performed using mouse-specific primers for EPC markers. Expression of mouse Flt-1 (mFlt-1), mouse Flk-1 (mFlk-1) and mouse CXCR4 (mCXCR4) in SL cells of PB were significantly greater in the pPlGF1 group than in the controls at day7 (**Figure S1*A*, *B*, *C***). Furthermore, double immunostaining for GFP and Sca-1 at day 7 after gene transfer revealed that pPlGF1 gene transfer resulted in recruiting two- to three-times the number of GFP^+^Sca-1^+^ cells into the peri-infarct borders (**Figure 4** ***B,C***). To further confirm that PlGF was capable of chemoattracting PB-SL cells, we used a modified Boyden chamber assay. This analysis revealed that PlGF chemo-attracted circulating SL cells in a dose-dependent manner (**Figure 4** ***D***), with a comparable potency to that of VEGF or SDF-1 (not shown).

Even though the above findings indicate that hPlGF1 itself is capable of inducing the proliferation of BM-SL cells in the BM - mobilizing them from the BM into the PB and recruiting them to the infarcted myocardium through direct effects on these cells - we also considered whether hPlGF1 might indirectly stimulate BM-SL accumulation in the infarct. Since SDF-1 is a chemokine for CXCR4^+^ BM-derived progenitors and known to enhance the recruitment of BM-derived EPCs into ischemic tissue as well as to entrap angio-competent CXCR4^+^ myeloid cells in peripheral tissues [17], we considered the possibility of whether hPlGF1 gene transfer upregulates SDF-1 expression in the ischemic myocardium. Indeed, real-time RT-PCR analysis revealed that rSDF-1 expression levels in the peri-infarct area were elevated more in the pPlGF1 group than in the controls (**Figure 4** ***E***). Immunostaining for SDF-1 at day 7 after gene transfer also revealed that the number of SDF-1^+^ cells was greater in the pPlGF1 group than the control groups (P<0.01 for pPlGF1 vs Mock and PBS) (**Figure 4** ***F–I***). Taken together, pPlGF1 transfer seemed to recruit BM-derived SL cells into the peri-infarct area either directly or indirectly through upregulation of the SDF-1/CXCR4 pathway.

### pPlGF gene transfer stimulates cardio-myogenesis from BM-SL cells

**Figure 5:**
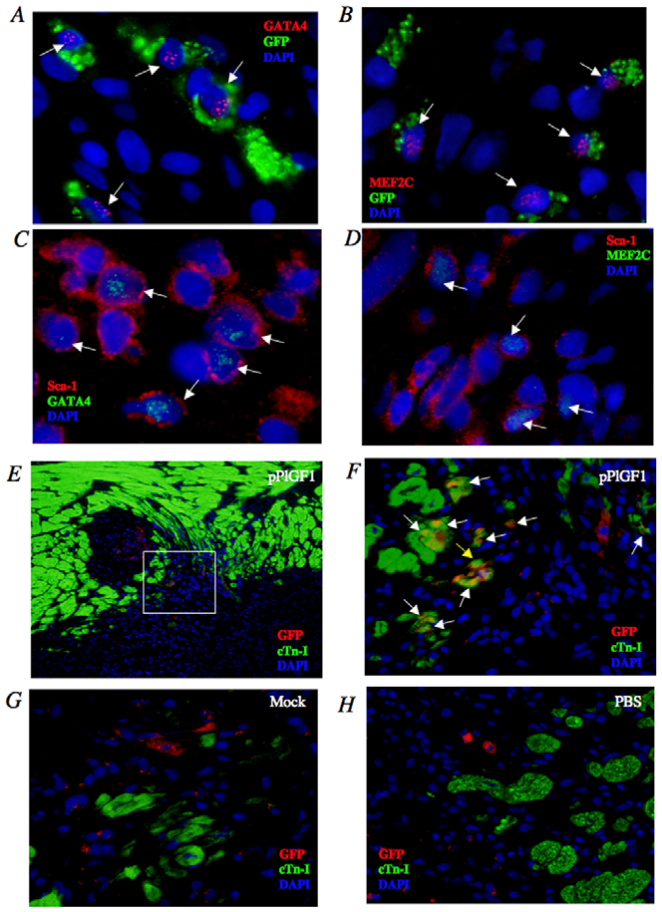
Representative double immunofluorescent staining for immature cardiomyogenic or vasculogenic markers and GFP or Sca-1 at day 7. ***A*** **,** ***B*** **,** Double immunofluorescent staining for GFP and GATA4 (***A***) or MEF2C (***B***) at day 7. BM-derived immature CMCs were identified as cells positive for cytoplasmic GFP (green) and nuclear GATA4 (***A***) or MEF2C (***B***) following pPlGF1 transfer. White arrows show nuclei of immature BM-derived CMCs (***A*** and ***B***). ***C*** **,** ***D***, Double immunofluorescent staining for Sca-1 and GATA4 (***C***) or MEF2C (***D***) at day 7. BM-derived immature CMCs were identified as cells positive for Sca-1 and nuclear GATA4 (green) (***C***) or MEF2C (green) (***D***) following pPlGF1 transfer. White arrows show nuclei of immature BM-derived CMCs (***C*** and ***D***). (n = 6 in each group). **Histological evaluation of development of BM-derived stem/progenitor cells into CMCs in rat ischemic myocardium at day 28.** ***E*** **,** ***F*** **:** Representative double immunofluorescent staining for cTn-I and GFP at day 28. BM-derived CMCs were identified as double positive cells for cTn-I (green) and GFP. *E*, merge in pPlGF1 group, ×10; *F*, merge in pPlGF1 group, ×40. White arrows show nuclei of BM-derived CMCs. ***G*** **,** ***H***: Representative double immunofluorescent staining for cardiac troponin-I (cTn-I) and GFP in Mock and PBS groups at day 28. BM-derived CMCs were identified as double positive cells for cTn-I (green) and GFP (red). *G,* merge in Mock group, ×40; *H*, merge in PBS group, ×40. (n = 6 in each group).

We also analyzed the cell fate of the BM-SL cells that were recruited to the peri-infarct borders by pPlGF1 gene transfer, and assessed whether they contributed to myocardial or vascular regeneration. At 7 days after gene transfer in BMT/MI/GTx rats, cells of the CMC lineage were identified by double immunostaining for GFP and GATA4, or its downstream target gene MEF2C, which is an established early cardiomyogenic marker. This analysis revealed that a small fraction of GFP^+^ cells in the peri-infarct borders co-expressed GATA4 or MEF2C in the animals that had received intramyocardial pPlGF1 gene transfer; in contrast, such marrow-derived CMCs expressing GFP were never or only very rarely detected in the control groups (**Figure 5** ***A, B***). These CMCs appeared morphologically mature and were indistinguishable from host CMCs in the peri-infarct and infarct area. Notably, GATA4^+^ and MEF2C^+^ cells were also found to express the progenitor marker Sca-1 (**Figure 5** ***C, D***), suggesting that PlGF1 gene transfer induced BM-derived SL progenitor cells to acquire the characteristics of the CMC lineage. This was further supported by the finding that certain GFP^+^ cells also expressed cTn-I (a marker of mature CMCs) at 28 days after gene transfer (**Figure 5** ***E,F***). Mock and PBS did not switch on CMC marker expression in GFP^+^ BM-SL cells (**Figure 5G,** ***H***). These findings suggest that BM-derived cell have the potential to differentiate into mature CMCs after PlGF gene transfer into infarcted myocardium. Frequency of the BM-derived CMCs (GFP+/cTn-I+ cells) to total BM-derived cells (GFP+ cells) was 11.8% in PlGF groups. In fact, 11.8% of newly formed CMCs derived from BM cells in PlGF group were developed through transdifferentiation mechanism between rat and mouse cells, whereas no transdifferentiation was observed in other groups.

GFP^+^ BM-SL cells in the pPlGF1 group were expressed connexin-43 with cTn-I and integrated with host myocardium through connexin-43. These findings demonstrated that PlGF gene therapy was one of the mechanism of restoring the loss of LV function (**Figure S2** ***A–D***).

### Supporting information Result

See Results S1 (**Figure S5, S6, S7, S8, S9, S10, S11, S12, S13**), which are published as supporting information on the PLos ONE web site.

## Discussion

In this paper, we show that myocardial PlGF gene transfer is a promising novel therapeutic strategy to revascularize and regenerate the infarcted myocardium and improve its performance after MI. Besides enhancing angiogenesis, PlGF induced this beneficial effect via two novel mechanisms, i.e. stimulation of cardiomyocyte survival, and induction of cardiac myoangiogenesis by BM progenitor cells, recruited to the infarct (**Figure S3**). To the best of our knowledge, this is the first report to document such a therapeutic potential and these novel mechanisms of PlGF gene therapy for ischemic heart disease.

Despite its discovery some 15 years ago now [1], the precise biological role of PlGF has remained puzzling for more than a decade. Over the last few years, however, PlGF has been increasingly recognized as contributing to the angiogenic switch in numerous disorders [1]–[5], [18]. In addition, transgenic overexpression or viral gene delivery of PlGF, or administration of recombinant PlGF enhance angiogenesis and vessel maturation in the skin and ischemic limb, thus indicating that PlGF has sufficient angiogenic potency [2], [4], [6], [7]. In this study, we found that PlGF and VEGFR1 expression levels were upregulated in the ischemic myocardium in rats, consistent with previous findings in mice and humans [2], [3], [7]. Injection of pPlGF1 in the ischemic myocardium further elevated PlGF levels for period of at least 7 days in vivo. Interestingly, this burst of PlGF secondarily amplified its own expression and induced the expression of several other angiogenic factors (VEGF, Ang1 and Ang2). PlGF may have induced the release of such angiogenic signals not only from resident cardiac cells but, perhaps, also from recruited BM-derived progenitors, similar to its effect on angiocompetent BM-derived VEGFR-1^+^ hemangiocytes, as previously documented [19]. This coordinated joint expression of paracrine angiogenic factors over a period of 7–10 days after MI sufficed to stimulate the formation of microvessels (endogenous angiogenesis) as well as of larger conduit vessels (endogenous arteriogenesis) for up to at least 28 days after MI. PlGF gene transfer thus stimulated the formation of durable, mature vessels, that failed to regress upon cessation of PlGF gene expression. These findings in the ischemic heart in the rat extend those previous findings in the skin and ischemic limb in mice that showed that PlGF promotes not only the formation of naked endothelial-lined channels but also recruits pericytes and smooth muscle cells around these nascent neovessels, thereby stabilizing them into more mature and regression-resistant vessels [3]. Importantly, this neovasculature was functional, as it improved cardiac performance after PlGF gene therapy.

An intriguing but medically relevant finding was that the infarct size was substantially reduced by myocardial PlGF gene therapy. Since the standardized procedure of LAD ligation results in a comparable at-risk ischemic myocardial area, and severely ischemic cardiomyocytes die within 24 to 48 hours (i.e. before new vessels are formed and are able to supply oxygen again), these findings suggest that PlGF gene transfer preserved cardiomyocyte viability initially via angiogenesis-independent mechanisms. In fact, echocardiography on day 5 revealed that LV functional parameters tended to be slightly better preserved in PlGF group than control groups although the difference was not statistically significant (FS: PlGF group, 21.6±1.6; Empty group, 19.1±1.6; PBS group, 20.1±2.0%; P = 0.053) (RWMS: PlGF group, 25.7±1.3; Empty group, 27.1±1.1; PBS group, 26.1±0.7%; P = 0.058). We cannot exclude the possibility that PlGF gene transfer rescued some of these ischemic myocytes in the peri-infarct border regions through vasodilation of residual vessels, but PlGF is a less potent vasodilator than VEGF (data not shown) [20]. Instead, PlGF gene transfer significantly reduced cardiomyocyte apoptosis, presumably through various complementary mechanisms. PlGF is a survival signal for endothelial cells and macrophages [9], but a similar activity of repairing endangered cardiomyocytes has not as yet been demonstrated. An intriguing question, however, is whether PlGF stimulates cardiomyocyte survival directly or indirectly. Though VEGFR1 is expressed on cardiomyocytes ([21] and unpublished observations), PlGF also stimulates the paracrine release of IGF-1 by cardiac fibroblasts, which express VEGFR-1 [3], [22], [23]. IGF-1 is well known for its effect on myocardial tissue, including stimulation of cardiac contractility, antiapoptotic effects and improved remodeling after MI by switching on a cascade of downstream pathways including activation of pAkt, MAPK and Bcl-xL [24], [25]. Moreover, PlGF also upregulated the expression of VEGF and Ang-1 [26], which have similar cardioprotective activity [16]. Furthermore, PlGF gene transfer augmented the accumulation of BM progenitors and endothelial cells, which release cardioprotective signals [27], [28]. The improved cardiac repair, and resultant smaller infarct size, likely contributed to the augmented cardiac performance after PlGF gene therapy.

Previous studies reported that VEGF is capable of recruiting angiocompetent VEGFR-1^+^ progenitors from the BM to sites of tissue regeneration or malignant growth [11], [19]. Though PlGF is known to stimulate cycling and differentiation of VEGFR-1^+^ Sca-1^+^lin^−^ (SL) cells after myeloablation [29] and to recruit BM-derived endothelial progenitors to tumors [10], the effects of PlGF on marrow progenitors have generally been much less characterized – especially in conditions of myocardial ischemia. We observed that PlGF has pleiotropic effects on BM-derived SL progenitors: (i) in vitro, PlGF dose-dependently stimulated the proliferation of SL progenitors, harvested from the BM, and was also capable of chemo-attracting SL cells, that were mobilized in the PB; (ii) in vivo, myocardial PlGF gene transfer increased the fraction of GFP^+^Sca-1^+^lin^−^ (GSL) cells in the BM and recruited these progenitors to the PB and, subsequently, in the peri-infarct borders in the heart. Apart from serving as a recruitment signal itself, PlGF also indirectly recruited BM progenitors to the ischemic heart. Indeed, similar to VEGF [19], PlGF upregulated myocardial expression of SDF-1, a well known chemo-attractant and retention signal of angiocompetent BM progenitors [19], [21]. Thus, myocardial PlGF gene therapy recruited marrow progenitors to the ischemic myocardium both via direct and indirect paracrine effects.

Consistent with previous findings [30], BM-derived GFP^+^ CMCs were only rarely detected in the peri-infarct borders in control animals after acute MI, suggesting that cardiomyogenesis is indeed a rare event. An appealing finding, therefore, is that intramyocardial PlGF gene transfer switched on the expression of CMC markers – initially of early (GATA-4, MEF-2C) and subsequently of mature (c-TnI) CMC markers – in at least a fraction of recruited progenitors. This is remarkable, as only a limited number of genes are thus far known to induce cardiomyogenesis of endogenous BM progenitors [31]. Indeed, many more studies have reported that BM-derived cells, upon intramyocardial implantation in infarcted hearts, can acquire functional and structural CMC characteristics without, however, identifying the responsible cardiomyogenic signals. Although VEGF promotes cardiomyogenic differentiation of embryonic stem cells through activation of Flk-1 and Flt-1 [8], the role of VEGF and PlGF in postnatal cardiomyogenesis has not been fully investigated. In vitro culture of BM Sca-1+/Lin- cells, with or without VEGF and PlGF, revealed that the combination of a higher concentration of PlGF and VEGF, but neither protein alone, is essential for early cardiac gene expression in BM stem cells (**Figure S4**). These results suggest that only PlGF protein may not directly induce cardiomyogenic differentiation of the BM stem cells. Certain combined signals from ischemic myocardium evoked by PlGF gene therapy may be necessary for inducing cardiomyogenesis in vivo. The fact that the expression of several angiogenic factors, including VEGF, was augmented following PlGF gene therapy in vivo may also support this hypothesis. The relative importance of exogenous cardiomyogenesis versus alternative mechanisms (i.e. regeneration by cardiac progenitors or endogenous CMCs) or cardioprotection (i.e. inhibition of apoptosis of endangered CMCs) needs to be defined in the future.

In conclusion, myocardial PlGF gene transfer improves cardiac performance after acute MI via complementary multi-tasking, namely, stimulation of endogenous angiogenesis and arteriogenesis (through direct and indirect paracrine effects), promotion of exogenous vasculogenesis (through direct and SDF-1-mediated recruitment of BM-progenitors and induction of endothelial commitment), cardioprotection (through inhibition of CMC apoptosis) and regeneration (through cardiomyogenesis).

## Supporting Information

Figure S1 ***A*** **,** ***B*** **,** ***C:*** Real-time PCR using PB-Sca-1^+^/lin^−^ (SL) cells isolated from BMT/MI/GTx rats in each group. pPlGF1 gene transfer significantly enhanced expression of mFlt-1, mCXCR4 and mFlk-1 mRNA in PB-SL cells 7 days after MI compared with controls. **, P<0.01 vs PBS; *, P<0.05 vs PBS; ††, P<0.01 vs Mock; †, P<0.05 vs Mock. (n = 8 in each group).
(TIF)

Figure S2 ***A*** **-** ***D:*** Representative immunofluorescent staining for cardiac troponin-I (cTn-I), GFP and connexin-43 in Mock, PBS and PlGF groups at day 28. BM-derived cardiomyogenic cells integrating with host cardiomyocytes were identified as triple positive cells for connexin-43 (red), cTn-I (blue) and GFP (green). *A*, merge in PBS group, ×20; *B*, merge in Mock group, ×20. *C*, merge in PlGF group, ×20; *D*, merge in PlGF group, higher magnification. White arrows show integration of the BM-derived cells with host cardiomyocytes. (n = 5 in each group).
(TIF)

Figure S3 Schema of therapeutic mechanisms of local gene transfer of PlGF for cardiac myoangiogenesis.
(TIF)

Figure S4 **PlGF combined with VEGF stimulates the expression of dexamethasone-induced myoangiogenic genes in BM stem cells in culture.** Real-time PCR for GATA4 (**A**), cTnI (**B**), Flt-1 (**C**), eNOS (**D**) and VE-cadherin (**E**) in BM Sca-1+/Lin− cells in culture. Left panels, mRNA expressions in BM Sca-1+/Lin− cells cultured with PlGF (0, 1, 10 or 100 ng/ml); Right panels, mRNA expressions in BM Sca-1+/Lin− cells cultured with VEGF (1 ng/ml) and PlGF (0, 1, 10 or 100 ng/ml). *, P<0.05 vs PlGF 0; †, P<0.05 vs PlGF 1. (n = 8 in each group).
(TIF)

Figure S5 ***A*** **:** Immunoblotting for hPlGF protein in cell lysate and culture medium of rat cardiomyoblasts (H9C2) 48 hours after transfection of IRESeGFP, phPlGF1-IRESeGFP and cDNA3 only. H9C2 without any transfection was used as negative control and recombinant hPlGF was prepared as positive control. hPlGF protein expression was detected in both cell lysate and medium following PlGF1-IRESeGFP transfer but not after control plasmid transfection. **PlGF signaling pathway is activated following MI.** ***B*** **:** Immunostaining for GFP using infarcted heart samples 4 days after pcDNA3-PlGF1–IRESeGFP plasmid or PBS injection (×10). ***C*** **:** Gene expression of hPlGF in MI tissue was detected at days 3 and 7 but not at day 14. lane 1, human heart (positive control); lane 2, PBS at day 3; lane 3, Mock at day 3; lane 4, phPlGF1 at day 3: lane 5, PBS at day 7; lane 6, Mock at day 7; lane 7, phPlGF1 at day 7: lane 8, PBS at day 14; lane 9, Mock at day 14; lane 10, phPlGF1 at day 14. (n = 5 in each group). ***D*** **,** ***E*** **:** ELISA revealed that hPlGF (***D***) and rVEGF (***E***) protein were detectable in plasma of PB 4 days post pPlGF1 gene transfer, but not in control groups. **, P<0.01 vs PBS; ††, P<0.01 vs Mock. (n = 5 in each group). ***F*** **,** ***G*** **:** PlGF signaling pathway is activated following myocardial infarction (MI). Expression of rPlGF (***F***) and its receptor rFlt-1 (***G***) mRNA 4 days after myocardial ischemia are upregulated. **, P<0.01. (n = 5 in each group).
(TIF)

Figure S6 **Representative double immunofluorescent staining for immature cardiomyogenic or vasculogenic markers and GFP or Sca-1 at day 7.** ***A*** **,** ***B*** **:** Double immunofluorescent staining for GFP and GATA6 (***A***) or Ets-1 (***B***) at day 7. BM-derived immature SMCs were detected as cells positive for cyoplasmic GFP and nuclear GATA6 (***A***) and the immature ECs were positive for GFP and Ets-1 (***B***). White arrows show nuclei of BM-derived immature SMCs (***A***) or ECs (***B***). ***C*** **,** ***D:*** Double immunofluorescent staining for Sca-1 and GATA6 (***C***) or Ets-1 (***D***) at day 7. BM-derived immature SMCs were detected as cells positive for Sca-1 and nuclear GATA6 (***C*** *)* and the immature ECs were positive for Sca-1 and Ets-1 (***D***). White arrows show nuclei of BM-derived immature SMCs (***C***) or ECs (***D***). (n = 6 in each group).
(TIF)

Figure S7 **Histological evaluation of development of BM-derived stem/progenitor cells into SMCs or ECs in rat ischemic myocardium at day 28.** ***A*** **,** ***B*** **:** Representative double immunofluorescent staining for smooth muscle actin (SMA) and GFP at day 28. BM-derived SMCs were identified as double positive cells for SMA and GFP (green). *A*, merge in pPlGF1 group, ×10; *B*, merge in pPlGF1 group, ×40. White arrows show nuclei of BM-derived SMCs. ***C*** **,** ***D*** **:** Representative double immunofluorescent staining for smooth muscle actin (SMA) and GFP in Mock and PBS groups at day 28. BM-derived SMCs were identified as double positive cells for SMA (red) and GFP (green). *C*, merge in Mock group, ×40; *D*, merge in PBS group, ×40. ***E*** **,** ***F*** **:** Representative double immunofluorescent staining for isolectin B4 (ILB4) (green) and GFP at day 28. BM-derived ECs were identified as double positive cells for ILB4 and GFP. *E*, merge in pPlGF1 group, ×10; *F*, merge in pPlGF1 group, ×40. White arrows show nuclei of BM-derived ECs. ***G*** **,** ***H*** **:** Representative double immunofluorescent staining for isolectin B4 (ILB4) (green) and GFP (red) in Mock and PBS groups at day 28. *G*, merge in Mock group, ×40; *H*, merge in PBS group, ×40. (n = 6 in each group).
(TIF)

Figure S8 LV functional evaluation by echocardiography and a micro-tip conductance catheter. Invasive hemodynamic parameters 28 days after pPlGF1, Mock or PBS injection. +dP/dt and −dP/dt, maximum and minimum derivative of LV pressure. ** or ††, P<0.01 vs PBS or Mock; †, P<0.05 vs Mock. (n = 8 in each group).
(TIF)

Figure S9 **pPlGF1 upregulates proliferation and survival of the BM progenitors.** ***A*** **:** BrdU incorporation revealed dose-dependent increase in proliferative activity of BM-SL cells after adding PlGF protein. *, P<0.05 vs 0 ng/ml; **, P<0.01 vs 0 ng/ml. ***B*** **:** The number of BM-SL cells in each well 1 week after cultivation increased by adding PlGF in a dose-dependent manner. *, P<0.05 vs 0 ng/ml; **, P<0.01 vs 0 ng/ml. (n = 6 in each group).
(TIF)

Figure S10 **pPlGF1 upregulates proliferation and survival of the BM progenitors.** ***A*** **:** The number of BM total MNCs obtained from xeno-rats was greater in the pPlGF1 group than controls. **, P<0.01 vs PBS; ††, P<0.01 vs Mock. ***B*** **:** Representative examples of serial FACS analysis of BM-GFP^+^/lin^−^ cells of xeno-rats in each group. Yellow colored population indicated GFP+/Sca-1+/lineage- (GSL) cells in BM of xeno-rats. **, P<0.01 vs PBS; ††, P<0.01 vs Mock. ***C*** **:** Percent of GSL cells in BM 7 and 28 days after MI was significantly increased in the pPlGF1 group than controls. **, P<0.01 vs PBS; ††, P<0.01 vs Mock. ***D*** **:** Immunoblotting for Akt, phospho-Akt and phospho-p44 or p42 MAPK in BM-SL cells 7 days after MI. Expression of these proteins was upregulated more in the pPlGF1 group than controls. (n = 6 in each group).
(TIF)

Figure S11 **pPlGF1 enhances cell growth-related signals in ischemic myocardium (endothelial cells).** **A:** Double immunofluorecent staining for phospho-Akt (pAkt, green) and isolectin B4 (ILB4, red). Double positive cells (white arrows) were identified as growing endothelial cells 7 days after administration of pPlGF1, Mock or PBS. **B:** Quantification of pAkt+/ILB4+ cells at peri-infarcted area in rats receiving pPlGF1, Mock or PBS at day 7. **, P<0.01 vs PBS; ††, P<0.01 vs Mock. **C:** Double Immunofluorecent staining for activated-MAPK (green) and IlB4 (red) at day 7. Double positive cells (white arrows) were identified as growing endothelial cells. **D:** Quantification of activated-MAPK+/ILB4+ cells at peri-infarcted area in rats receiving pPlGF1, Mock or PBS at day 7. **, P<0.01 vs PBS; ††, P<0.01 vs Mock. (n = 6 in each group).
(TIF)

Figure S12 **pPlGF1 enhances cell growth-related signals in ischemic myocardium (cardiomyocytes).** **A:** Double staining for activated-MAPK (green) and cardiac troponin I (cTnI, red) at day 7. Double positive cells (white arrowheads) were identified as growing cardiomyocytes. **B:** Quantification of activated-MAPK+/cTnI+ cells at peri-infarcted area in rats receiving pPlGF1, Mock or PBS at day 7. (n = 6 in each group).
(TIF)

Figure S13 ***A*** **:** Representative morphology of small and large endothelial progenitor cell (EPC) colonies. ***B*** **:** In EPC colony forming assay in BM-SL cells of xeno-rats, the number of small EPC colonies, large EPC colonies and total EPC colonies were significantly greater in the pPlGF1 group than controls. *, P<0.05 vs PBS; †, P<0.05 vs Mock. (n = 6 in each group).
(TIF)

Results S1 Supporting Information Results.
(DOC)
