# Supplementary figures and images for: PlGF Repairs Myocardial Ischemia through Mechanisms of Angiogenesis, Cardioprotection and Recruitment of Myo-Angiogenic Competent Marrow Progenitors
Source: PLoS One. 2011 Sep 28;6(9):e24872. doi: 10.1371/journal.pone.0024872 (PMC3182165; doi:10.1371/journal.pone.0024872)

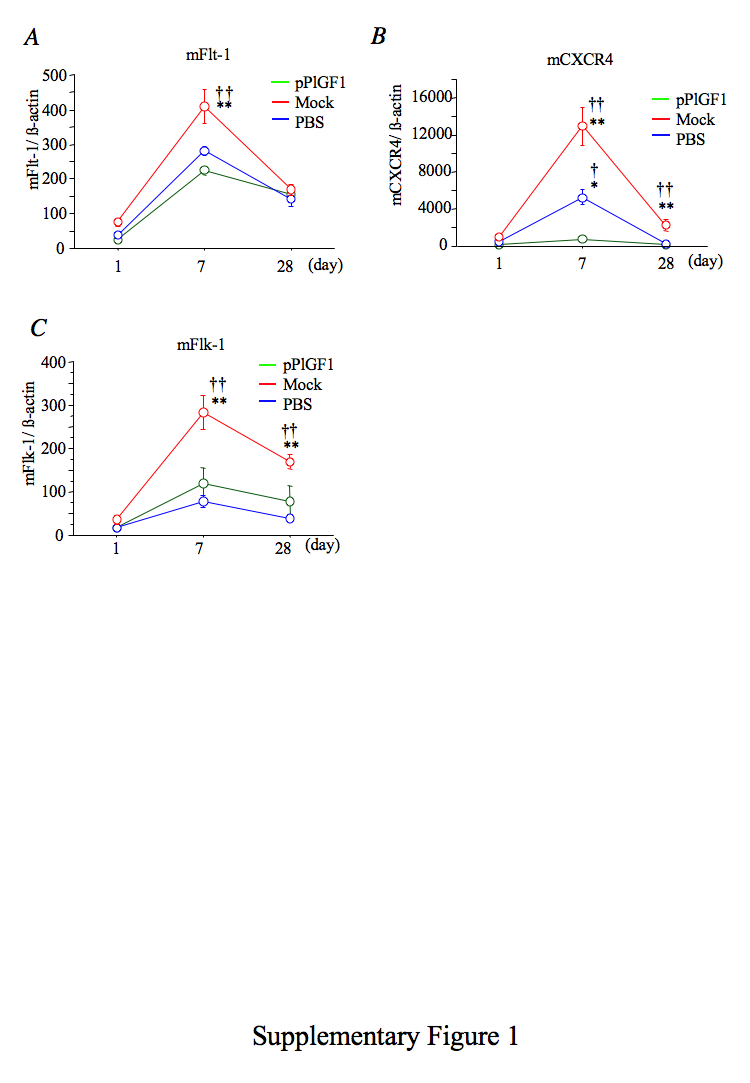

Supplement: Figure S1 — A , B , C: Real-time PCR using PB-Sca-1+/lin− (SL) cells isolated from BMT/MI/GTx rats in each group. pPlGF1 gene transfer significantly enhanced expression of mFlt-1, mCXCR4 and mFlk-1 mRNA in PB-SL cells 7 days after MI compared with controls. **, P<0.01 vs PBS; *, P<0.05 vs PBS; ††, P<0.01 vs Mock; †, P<0.05 vs Mock. (n = 8 in each group). (TIF) [file pone.0024872.s001.tif]

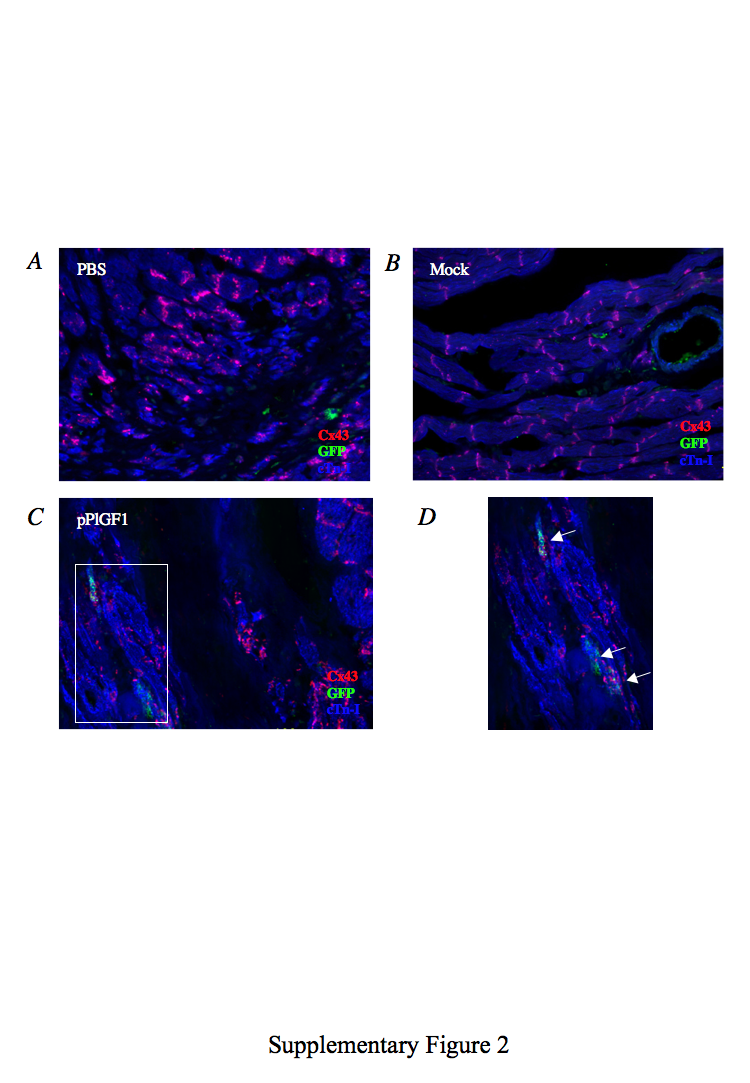

Supplement: Figure S2 — A - D: Representative immunofluorescent staining for cardiac troponin-I (cTn-I), GFP and connexin-43 in Mock, PBS and PlGF groups at day 28. BM-derived cardiomyogenic cells integrating with host cardiomyocytes were identified as triple positive cells for connexin-43 (red), cTn-I (blue) and GFP (green). A, merge in PBS group, ×20; B, merge in Mock group, ×20. C, merge in PlGF group, ×20; D, merge in PlGF group, higher magnification. White arrows show integration of the BM-derived cells with host cardiomyocytes. (n = 5 in each group). (TIF) [file pone.0024872.s002.tif]

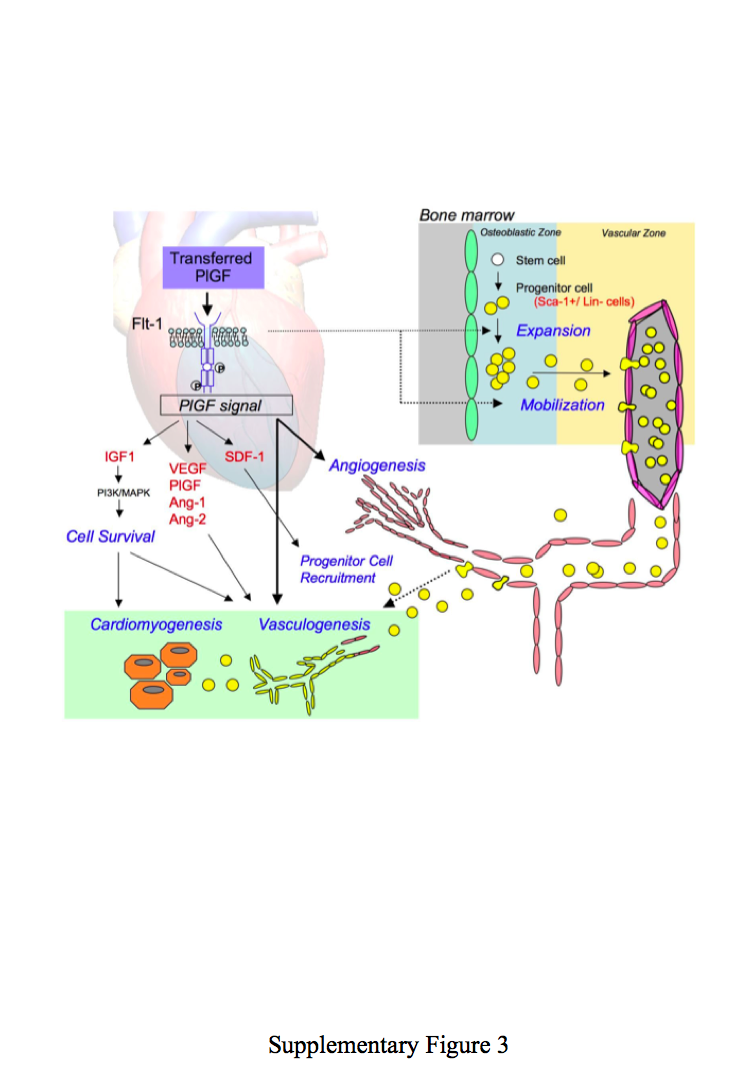

Supplement: Figure S3 — Schema of therapeutic mechanisms of local gene transfer of PlGF for cardiac myoangiogenesis. (TIF) [file pone.0024872.s003.tif]

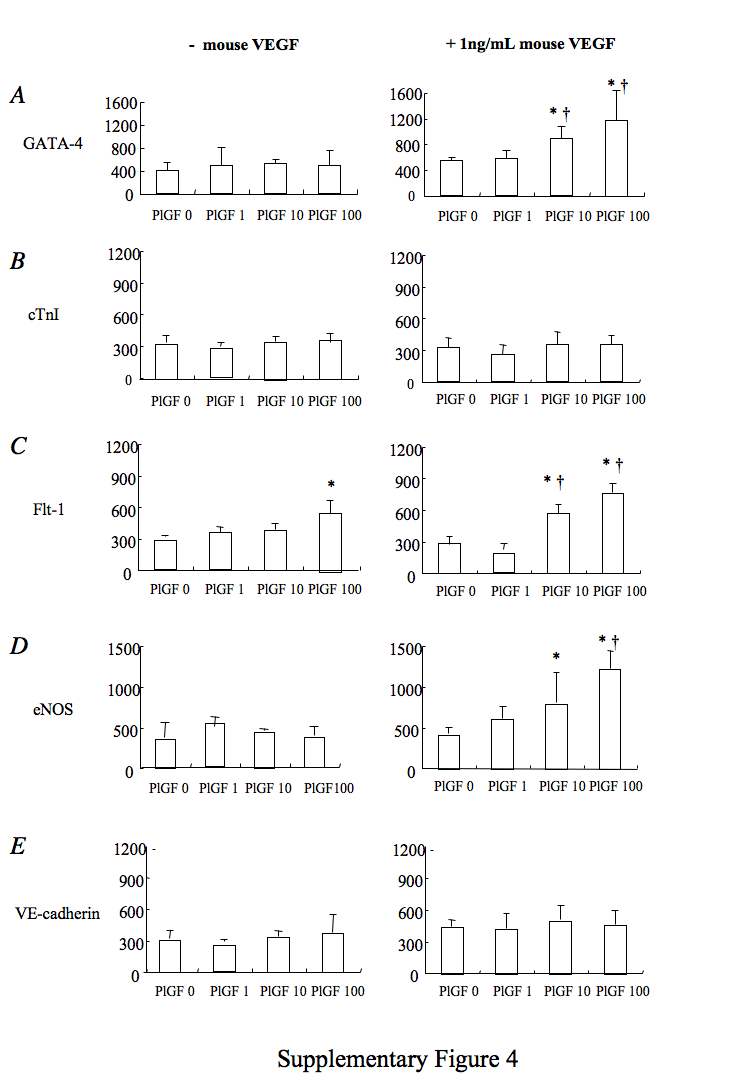

Supplement: Figure S4 — PlGF combined with VEGF stimulates the expression of dexamethasone-induced myoangiogenic genes in BM stem cells in culture. Real-time PCR for GATA4 (A), cTnI (B), Flt-1 (C), eNOS (D) and VE-cadherin (E) in BM Sca-1+/Lin− cells in culture. Left panels, mRNA expressions in BM Sca-1+/Lin− cells cultured with PlGF (0, 1, 10 or 100 ng/ml); Right panels, mRNA expressions in BM Sca-1+/Lin− cells cultured with VEGF (1 ng/ml) and PlGF (0, 1, 10 or 100 ng/ml). *, P<0.05 vs PlGF 0; †, P<0.05 vs PlGF 1. (n = 8 in each group). (TIF) [file pone.0024872.s004.tif]

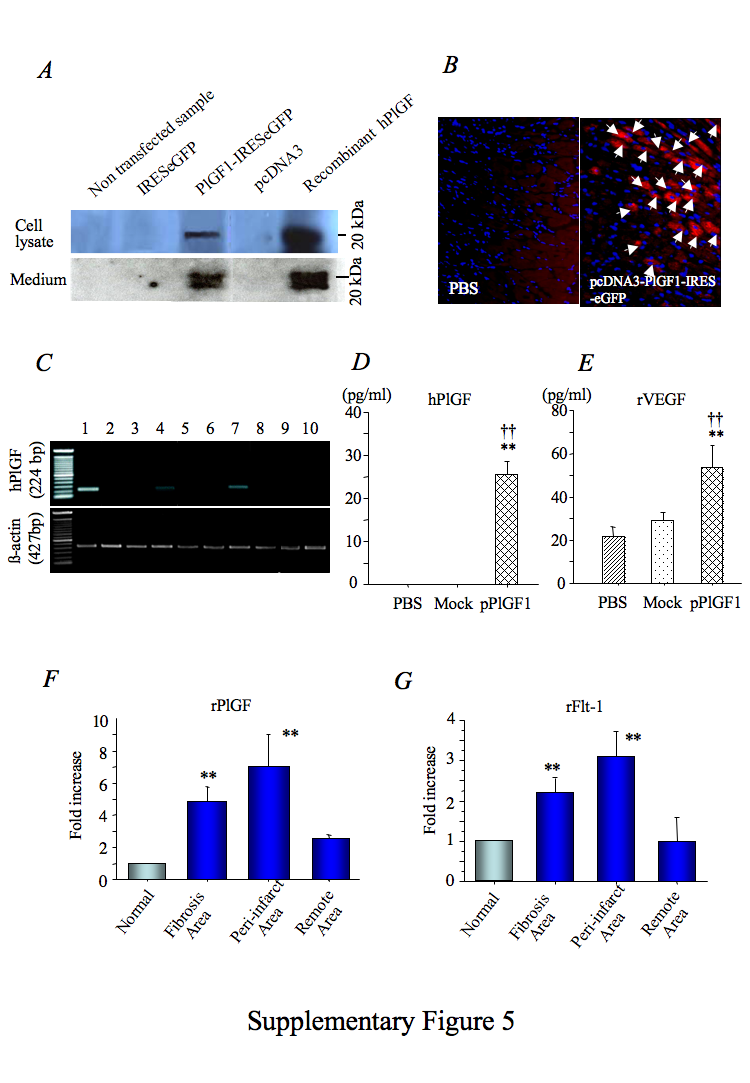

Supplement: Figure S5 — A : Immunoblotting for hPlGF protein in cell lysate and culture medium of rat cardiomyoblasts (H9C2) 48 hours after transfection of IRESeGFP, phPlGF1-IRESeGFP and cDNA3 only. H9C2 without any transfection was used as negative control and recombinant hPlGF was prepared as positive control. hPlGF protein expression was detected in both cell lysate and medium following PlGF1-IRESeGFP transfer but not after control plasmid transfection. PlGF signaling pathway is activated following MI. B : Immunostaining for GFP using infarcted heart samples 4 days after pcDNA3-PlGF1–IRESeGFP plasmid or PBS injection (×10). C : Gene expression of hPlGF in MI tissue was detected at days 3 and 7 but not at day 14. lane 1, human heart (positive control); lane 2, PBS at day 3; lane 3, Mock at day 3; lane 4, phPlGF1 at day 3: lane 5, PBS at day 7; lane 6, Mock at day 7; lane 7, phPlGF1 at day 7: lane 8, PBS at day 14; lane 9, Mock at day 14; lane 10, phPlGF1 at day 14. (n = 5 in each group). D , E : ELISA revealed that hPlGF (D) and rVEGF (E) protein were detectable in plasma of PB 4 days post pPlGF1 gene transfer, but not in control groups. **, P<0.01 vs PBS; ††, P<0.01 vs Mock. (n = 5 in each group). F , G : PlGF signaling pathway is activated following myocardial infarction (MI). Expression of rPlGF (F) and its receptor rFlt-1 (G) mRNA 4 days after myocardial ischemia are upregulated. **, P<0.01. (n = 5 in each group). (TIF) [file pone.0024872.s005.tif]

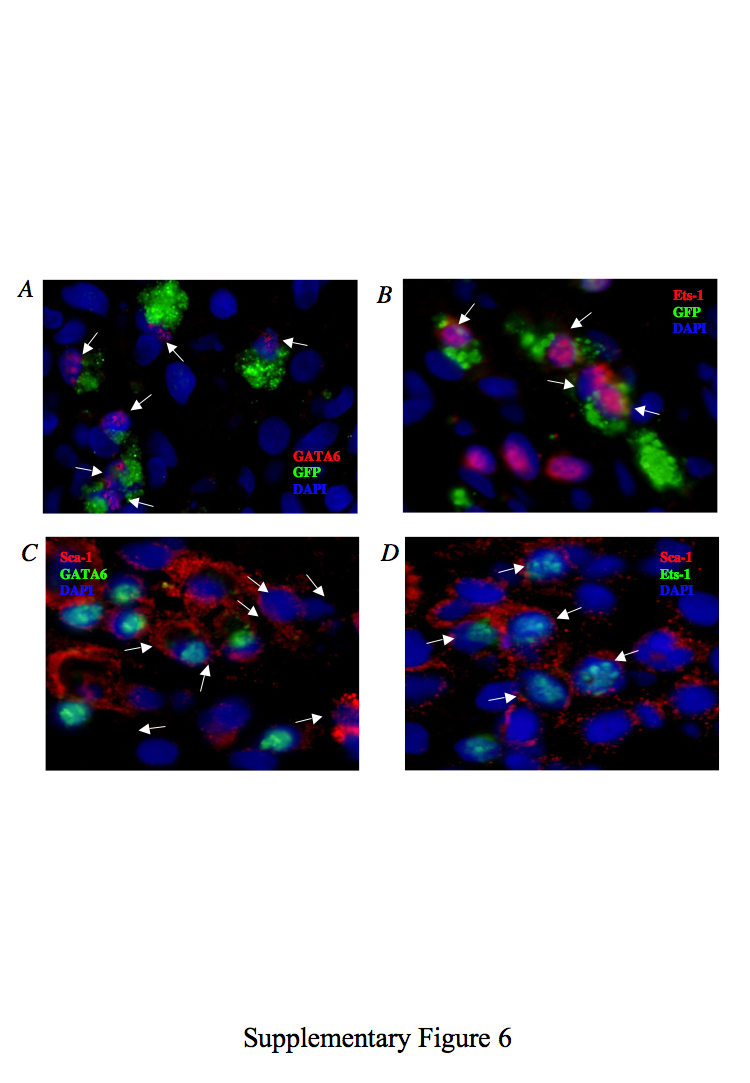

Supplement: Figure S6 — Representative double immunofluorescent staining for immature cardiomyogenic or vasculogenic markers and GFP or Sca-1 at day 7. A , B : Double immunofluorescent staining for GFP and GATA6 (A) or Ets-1 (B) at day 7. BM-derived immature SMCs were detected as cells positive for cyoplasmic GFP and nuclear GATA6 (A) and the immature ECs were positive for GFP and Ets-1 (B). White arrows show nuclei of BM-derived immature SMCs (A) or ECs (B). C , D: Double immunofluorescent staining for Sca-1 and GATA6 (C) or Ets-1 (D) at day 7. BM-derived immature SMCs were detected as cells positive for Sca-1 and nuclear GATA6 (C ) and the immature ECs were positive for Sca-1 and Ets-1 (D). White arrows show nuclei of BM-derived immature SMCs (C) or ECs (D). (n = 6 in each group). (TIF) [file pone.0024872.s006.tif]

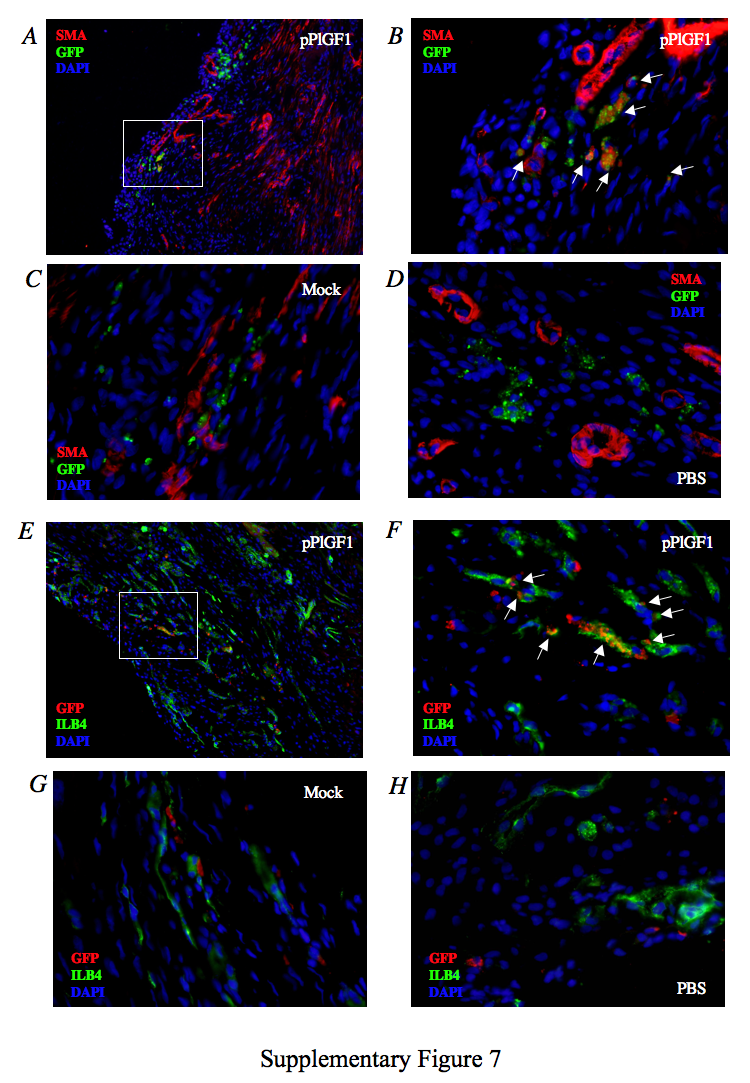

Supplement: Figure S7 — Histological evaluation of development of BM-derived stem/progenitor cells into SMCs or ECs in rat ischemic myocardium at day 28. A , B : Representative double immunofluorescent staining for smooth muscle actin (SMA) and GFP at day 28. BM-derived SMCs were identified as double positive cells for SMA and GFP (green). A, merge in pPlGF1 group, ×10; B, merge in pPlGF1 group, ×40. White arrows show nuclei of BM-derived SMCs. C , D : Representative double immunofluorescent staining for smooth muscle actin (SMA) and GFP in Mock and PBS groups at day 28. BM-derived SMCs were identified as double positive cells for SMA (red) and GFP (green). C, merge in Mock group, ×40; D, merge in PBS group, ×40. E , F : Representative double immunofluorescent staining for isolectin B4 (ILB4) (green) and GFP at day 28. BM-derived ECs were identified as double positive cells for ILB4 and GFP. E, merge in pPlGF1 group, ×10; F, merge in pPlGF1 group, ×40. White arrows show nuclei of BM-derived ECs. G , H : Representative double immunofluorescent staining for isolectin B4 (ILB4) (green) and GFP (red) in Mock and PBS groups at day 28. G, merge in Mock group, ×40; H, merge in PBS group, ×40. (n = 6 in each group). (TIF) [file pone.0024872.s007.tif]

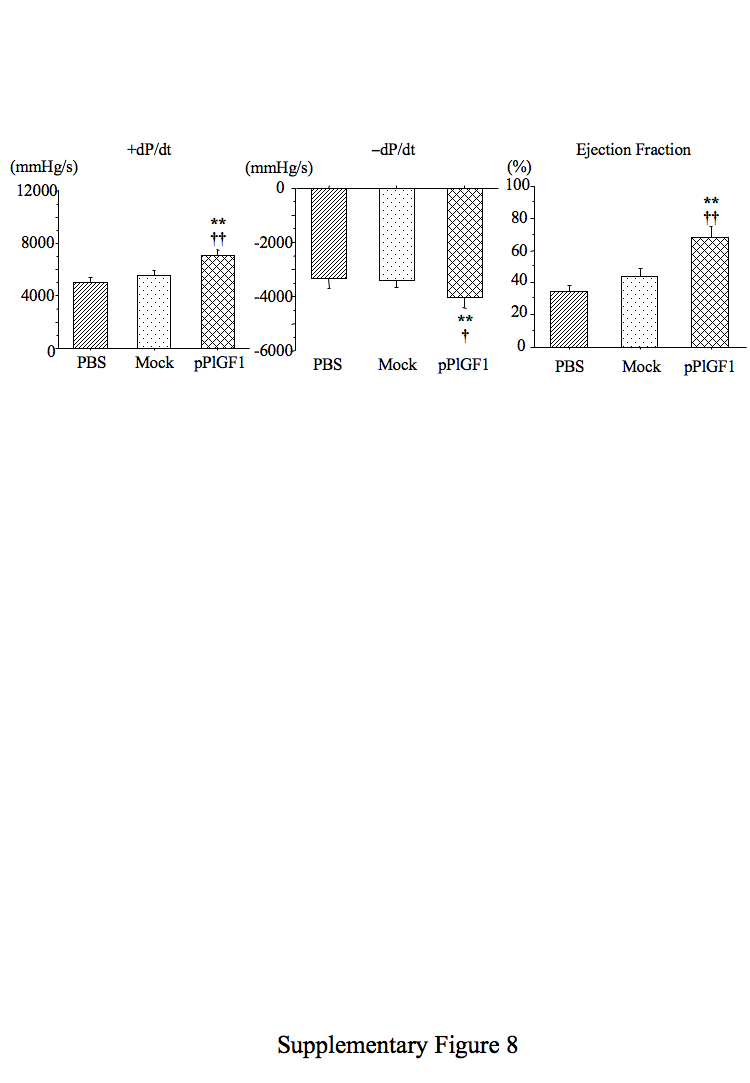

Supplement: Figure S8 — LV functional evaluation by echocardiography and a micro-tip conductance catheter. Invasive hemodynamic parameters 28 days after pPlGF1, Mock or PBS injection. +dP/dt and −dP/dt, maximum and minimum derivative of LV pressure. ** or ††, P<0.01 vs PBS or Mock; †, P<0.05 vs Mock. (n = 8 in each group). (TIF) [file pone.0024872.s008.tif]

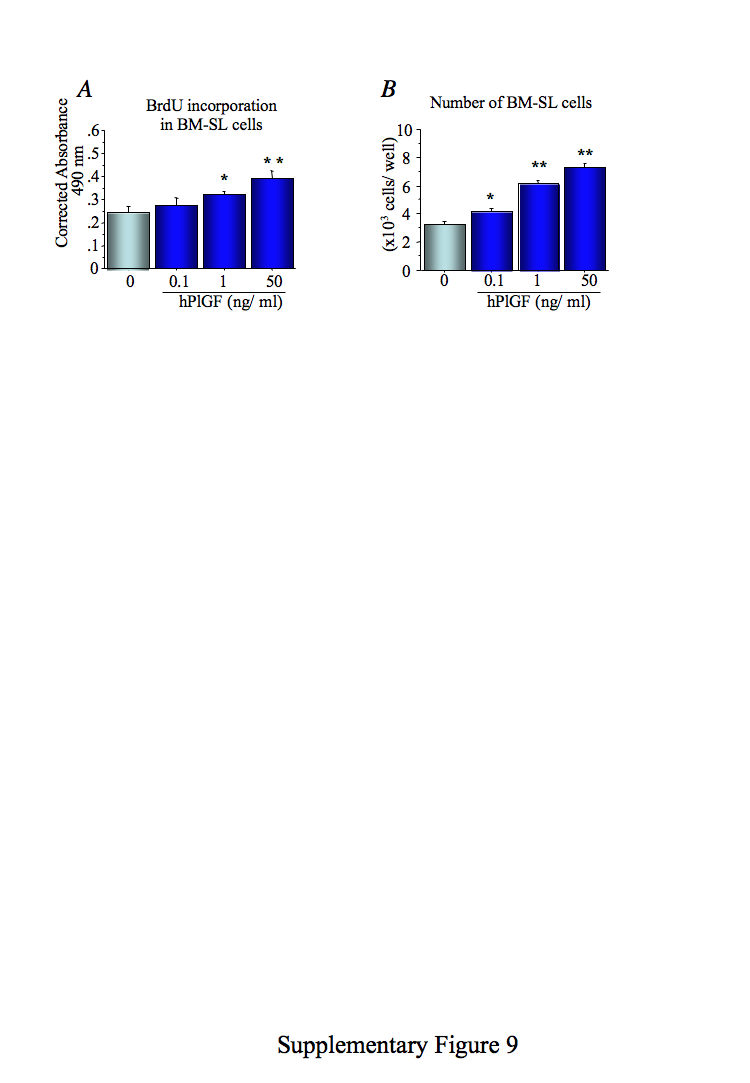

Supplement: Figure S9 — pPlGF1 upregulates proliferation and survival of the BM progenitors. A : BrdU incorporation revealed dose-dependent increase in proliferative activity of BM-SL cells after adding PlGF protein. *, P<0.05 vs 0 ng/ml; **, P<0.01 vs 0 ng/ml. B : The number of BM-SL cells in each well 1 week after cultivation increased by adding PlGF in a dose-dependent manner. *, P<0.05 vs 0 ng/ml; **, P<0.01 vs 0 ng/ml. (n = 6 in each group). (TIF) [file pone.0024872.s009.tif]

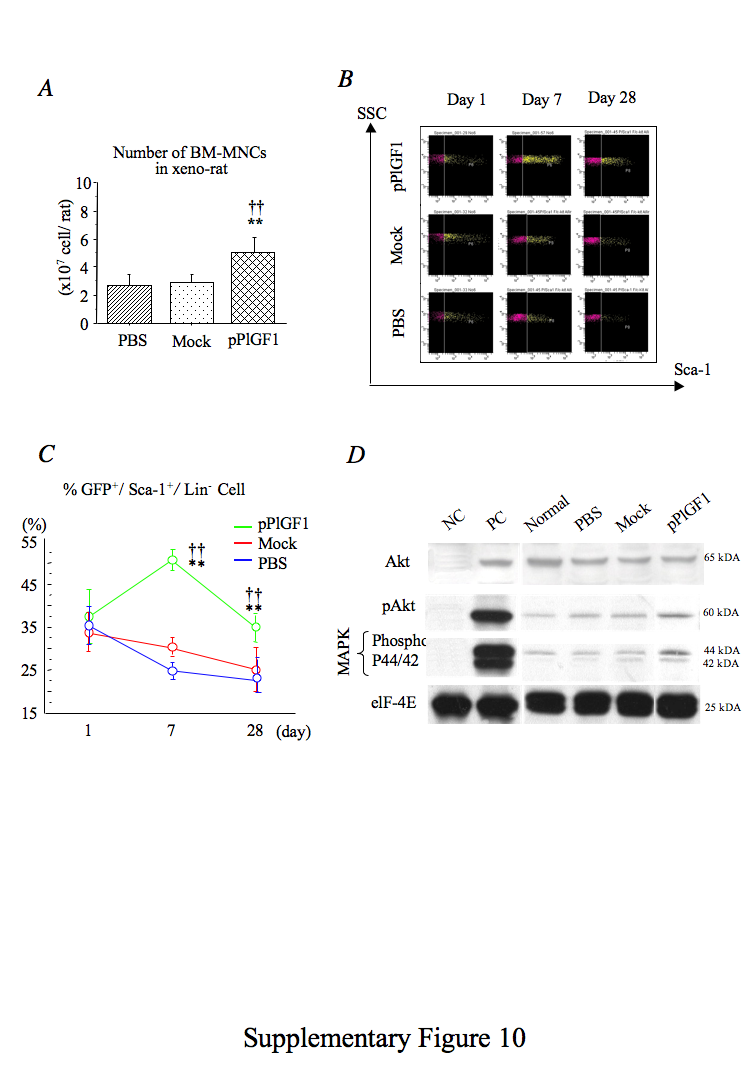

Supplement: Figure S10 — pPlGF1 upregulates proliferation and survival of the BM progenitors. A : The number of BM total MNCs obtained from xeno-rats was greater in the pPlGF1 group than controls. **, P<0.01 vs PBS; ††, P<0.01 vs Mock. B : Representative examples of serial FACS analysis of BM-GFP+/lin− cells of xeno-rats in each group. Yellow colored population indicated GFP+/Sca-1+/lineage- (GSL) cells in BM of xeno-rats. **, P<0.01 vs PBS; ††, P<0.01 vs Mock. C : Percent of GSL cells in BM 7 and 28 days after MI was significantly increased in the pPlGF1 group than controls. **, P<0.01 vs PBS; ††, P<0.01 vs Mock. D : Immunoblotting for Akt, phospho-Akt and phospho-p44 or p42 MAPK in BM-SL cells 7 days after MI. Expression of these proteins was upregulated more in the pPlGF1 group than controls. (n = 6 in each group). (TIF) [file pone.0024872.s010.tif]

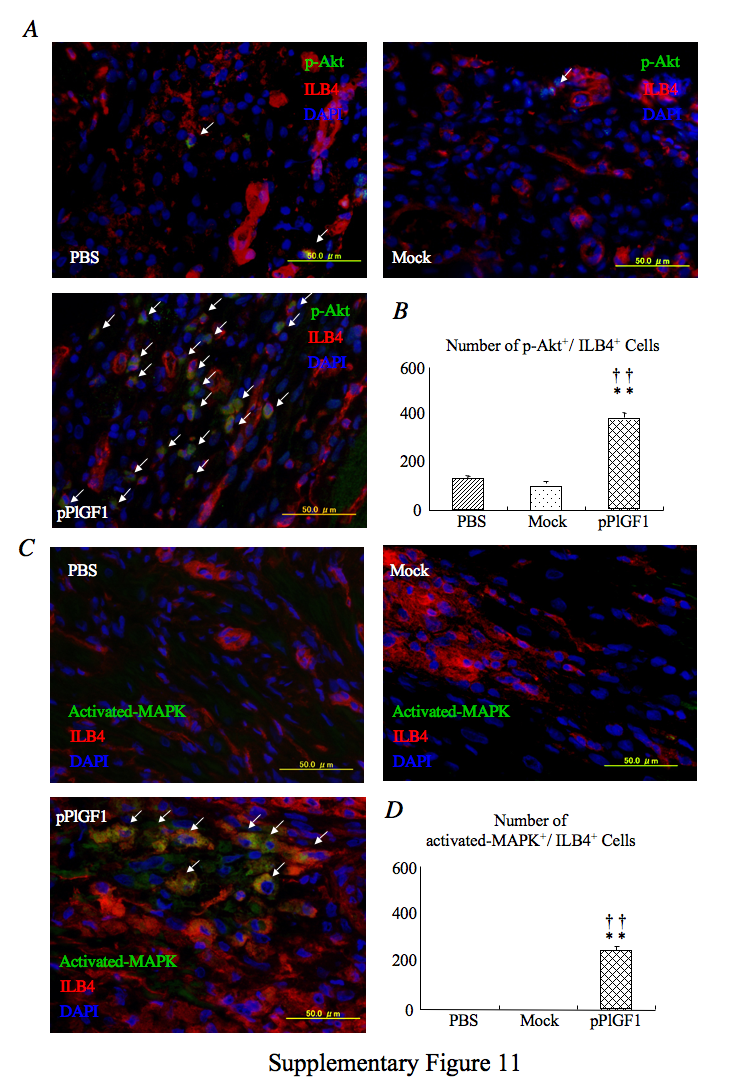

Supplement: Figure S11 — pPlGF1 enhances cell growth-related signals in ischemic myocardium (endothelial cells). A: Double immunofluorecent staining for phospho-Akt (pAkt, green) and isolectin B4 (ILB4, red). Double positive cells (white arrows) were identified as growing endothelial cells 7 days after administration of pPlGF1, Mock or PBS. B: Quantification of pAkt+/ILB4+ cells at peri-infarcted area in rats receiving pPlGF1, Mock or PBS at day 7. **, P<0.01 vs PBS; ††, P<0.01 vs Mock. C: Double Immunofluorecent staining for activated-MAPK (green) and IlB4 (red) at day 7. Double positive cells (white arrows) were identified as growing endothelial cells. D: Quantification of activated-MAPK+/ILB4+ cells at peri-infarcted area in rats receiving pPlGF1, Mock or PBS at day 7. **, P<0.01 vs PBS; ††, P<0.01 vs Mock. (n = 6 in each group). (TIF) [file pone.0024872.s011.tif]

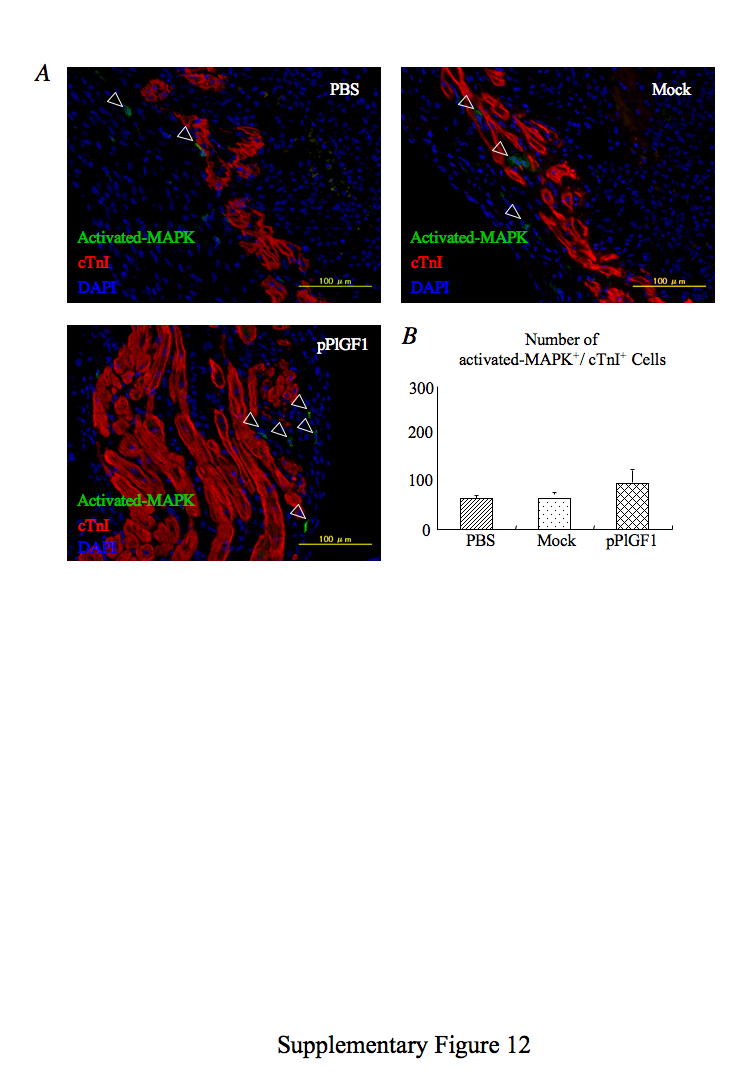

Supplement: Figure S12 — pPlGF1 enhances cell growth-related signals in ischemic myocardium (cardiomyocytes). A: Double staining for activated-MAPK (green) and cardiac troponin I (cTnI, red) at day 7. Double positive cells (white arrowheads) were identified as growing cardiomyocytes. B: Quantification of activated-MAPK+/cTnI+ cells at peri-infarcted area in rats receiving pPlGF1, Mock or PBS at day 7. (n = 6 in each group). (TIF) [file pone.0024872.s012.tif]

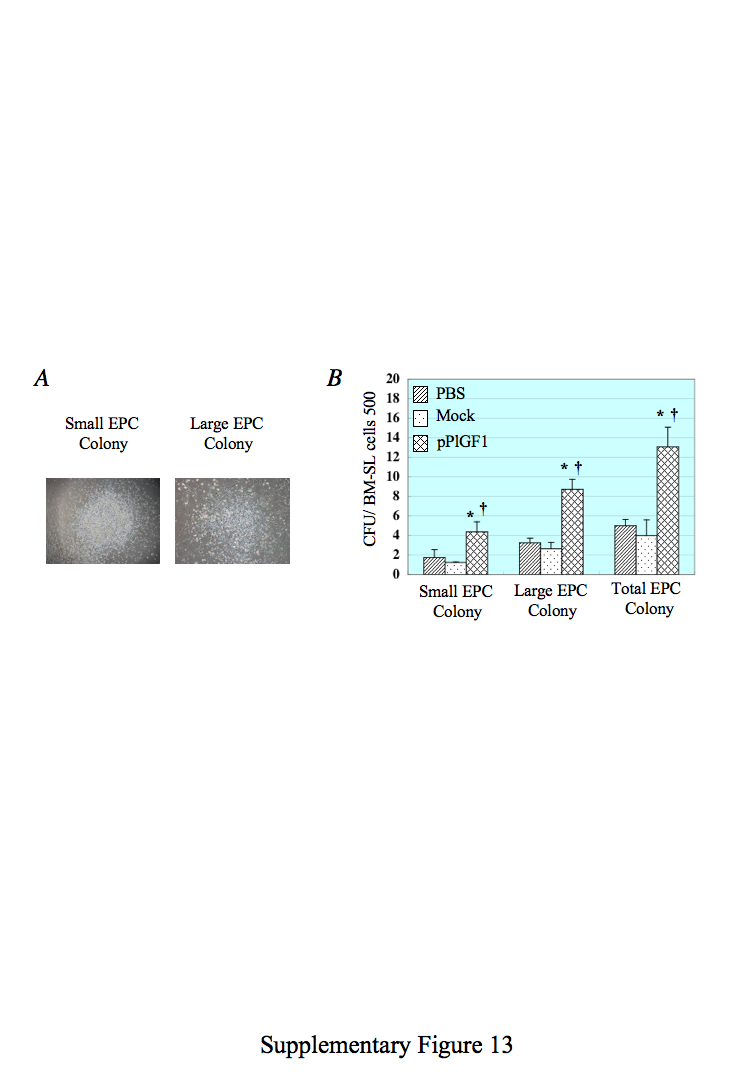

Supplement: Figure S13 — A : Representative morphology of small and large endothelial progenitor cell (EPC) colonies. B : In EPC colony forming assay in BM-SL cells of xeno-rats, the number of small EPC colonies, large EPC colonies and total EPC colonies were significantly greater in the pPlGF1 group than controls. *, P<0.05 vs PBS; †, P<0.05 vs Mock. (n = 6 in each group). (TIF) [file pone.0024872.s013.tif]
