# Supplementary material for: PlGF Repairs Myocardial Ischemia through Mechanisms of Angiogenesis, Cardioprotection and Recruitment of Myo-Angiogenic Competent Marrow Progenitors
Source: PLoS One. 2011 Sep 28;6(9):e24872. doi: 10.1371/journal.pone.0024872 (PMC3182165; doi:10.1371/journal.pone.0024872)
Supplement: Results S1 — Supporting Information Results. (DOC) [file pone.0024872.s014.doc]

**Supporting Information**

***Results***

**PlGF combined with VEGF enhances the expression of early cardiac and endothelial markers in BM stem cells *in vitro.***

We sought to determine the molecular mechanism by which PlGF directly or indirectly induces differentiation of BM stem cells into cardiomyocytes or endothelial cells. Cardiomyogenic and vasculogenic differentiation of BM Sca-1+/Lin- cells was induced by addition of dexamethasone. We performed real-time RT-PCR to quantify the transcript levels of GATA4, cTnI, Flt-1, eNOS and VE-cadherin in BM Sca-1^+^/Lin^-^ cells 7 days after culture with or without PlGF and VEGF. Addition of 0, 1, 10 or 100 ng/ml of PlGF only (without VEGF) or 1 ng/ml of VEGF only (without PlGF) did not significantly affect the mRNA expression levels in the BM stem cells. However, GATA4 and Flt-1mRNA levels were higher in the BM stem cells cultured with 10 or 100 ng/ml of PlGF and 1 ng/ml of VEGF than those with 0 or 1 ng/ml of PlGF and 1 ng/ml of VEGF. Real-time RT-PCR analysis also revealed that expression of eNOS but not VE-cadherin was greater in the BM cells with 100 ng/ml of PlGF and 1 ng/ml of VEGF than those with 0 or 1 ng/ml of PlGF and 1 ng/ml of VEGF. The expression of eNOS was also higher in the BM cells with 10 ng/ml of PlGF and 1 ng/ml of VEGF than those with 1 ng/ml of VEGF only **(Figure S4)**. These results indicate that the combination of PlGF and VEGF, but not either single growth factor alone, may be essential to induce cardiomyogenic and vasculogenic differentiation of BM stem cells *in vitro*.

**Plasmid transfer induces expression of human PlGF1 (*in vitro*)**

In order to assess whether PlGF gene transfer stimulates revascularization and regeneration of the post-MI ischemic myocardium, we constructed an expression vector of human PlGF1 (pPlGF1) [1]. Compared to PlGF2, this isoform of PlGF binds less to the extracellular matrix, is therefore more freely diffusible, and might thus be expected to have a wider action radius; it also circulates longer in the peripheral blood (several hours; unpublished)[1]. To confirm that pPlGF1 gene transfer increased hPlGF1 expression levels, we transfected this construct in the rat cardiomyoblast H9C2 cell line. Immunoblotting revealed detectable hPlGF1 protein levels in both the cell lysate and conditioned medium of the transfected cells, indicating that PlGF1 gene transfer indeed resulted in the production and release of hPlGF1 protein **(Figure S5*A*)**.

**Intra-myocardial plasmid transfer induces expression of human PlGF1**

To visualize the hPlGF1-expressing myocardial cells, we also constructed another plasmid, encoding both hPlGF1 and eGFP (pcDNA3-PlGF1-IRESeGFP). Intramyocardial injection into the ischemic border zone of 500 μg pcDNA3-PlGF1-IRESeGFP or an empty pcDNA3 control plasmid at 20 minutes after ligation of the LAD in Sprague Dawley rats resulted, 4 days later, in GFP expression in the pcDNA3-PlGF1-IRESeGFP but not in the control group **(Figure S5*B*)**. RT-PCR analysis revealed that, in the pPlGF1 group, hPlGF1 was transiently expressed in the infarcted myocardium at day 3 and 7, but no longer at day 14 post-MI **(Figure S5*C*)**. Furthermore, ELISA revealed that hPlGF1 was detectable in the peripheral blood at 4 days after gene transfer in the pPlGF1 group but not in the control groups (P<0.01 for pPlGF1 vs controls). Interestingly, plasma levels of rat VEGF (rVEGF) were significantly greater in the pPlGF1 group (P<0.01 for pPlGF1 vs controls) **(Figure S5*D*, *E*)**.

**PlGF and its receptor Flt-1 are upregulated in ischemic myocardium**

We first analyzed whether PlGF and its receptor Flt-1 are expressed in the ischemic myocardium. Real-time RT-PCR of rat hearts obtained at 4 days after ligation of the left anterior descending (LAD) coronary artery [2] revealed that mRNA expression of endogenous PlGF (rPlGF) increased 7.21 ± 0.53 fold in the peri-infarct area as compared to the non-ischemic myocardium **(Figure S5*F*)**. Transcript levels of the PlGF receptor Flt-1 (rFlt-1) were also increased 3.24 ± 0.41 fold in the peri-infarct area **(Figure S5*G*)**. These data suggest to us that PlGF is involved in the myocardial response to ischemia.

**pPlGF gene transfer stimulates vascular regeneration**

A similar analysis revealed that pPlGF1 gene transfer, but not Mock or PBS, changed the phenotype of a fraction of GFP^+^ and Sca-1^+^ cells, that were recruited in the ischemic myocardium, by inducing the expression of the immature endothelial marker Ets-1 and of the early SMC marker GATA6 **(Figure S6*A-D*)**. Subsequently, at 28 days after gene transfer, these cells co-expressed the mature EC marker isolectin-B4 or the SMC marker smooth muscle α-actin (SMA) **(Figure S7*A*-*H*)**.

**pPlGF1 improves left ventricular function after acute MI**

We also used high-fidelity micromanometer-tipped conductance catheters to analyze the hemodynamic performance of the LV at 4 weeks after gene transfer. Consistent with the echocardiographic analysis, pPlGF1 treatment improved LV pump function. Indeed, the +dP/dt (mmHg/sec), a measure of the contractility, was 5,820 ± 233 for Mock and 5,020 ± 233 for PBS, but 7,370 ± 111 for pPlGF1 (P<0.01 for pPlGF1 vs Mock and PBS). LV relaxation was also improved by pPlGF: the –dP/dt values (mmHg/sec) were –3,320 ± 233 for Mock, –3,210 ± 212 for PBS and –4,050 ± 170 for pPlGF1 (P<0.05 for pPlGF1 vs Mock, P<0.01 for pPlGF1 vs PBS). The ejection fraction (EF) was also improved by pPlGF: 41.2 ± 2.3% for Mock, 31.9 ± 1.4% for PBS and 68.5 ± 1.4% for pPlGF1 (P<0.01 for pPlGF1 vs Mock and PBS) **(Figure S8)**. None of these parameters showed significant differences between Mock and PBS groups at 4 weeks after gene transfer. The heart rate (HR) was also comparable in each group (not shown). Thus, transfer of pPlGF1 both preserved and improved global and regional LV function post-MI.

**PlGF stimulates the proliferation of Sca-1+/lin– (SL) progenitors *in vitro***

We also explored whether pPlGF1 gene transfer stimulated myocardial angiogenesis and regeneration post-MI by recruiting BM-derived progenitors. We therefore first analyzed whether PlGF affected the proliferation of BM-derived Sca-1^+^lin^–^ (BM-SL) progenitors *in vitro*, using a quantitative bromo-deoxyuridine (BrdU) incorporation assay. BM-SL cells were selected, as they are capable of differentiating into different cell lineages. Supplementation of hPlGF1 dose-dependently increased the proliferation of these BM-SL cells **(Figure S9*A*)**. A count of BM-SL cells confirmed that hPlGF1 increased the total number of these progenitors in a dose-dependent manner (P<0.05 for 0 vs 0.1 ng/ ml and P<0.01 for 0 vs 1 and 50 ng/ ml) **(Figure S9*B*)**. Thus, PlGF stimulates BM-SL expansion *in vitro*.

**pPlGF1 gene transfer stimulates BM-SL cell growth in infarcted rats**

To assess whether pPlGF1 gene transfer stimulated the expansion of BM-SL cells in vivo as well, we xeno-transplanted BM of transgenic mice, ubiquitously expressing green fluorescent protein (GFP), into sublethally irradiated nude rats (xeno-rats), which enabled us to count the number of BM-derived GFP^+^ cells. After recovery from the BM xeno-transplantation (BMT) and reconstitution of the hematopoietic system, we ligated the coronary LAD in these xeno-rats, and injected them intra-myocardially with pPlGF, Mock or PBS. Infarcted xeno-rats that received gene transfer (GTx) are referred to as BMT/MI/GTx animals from here on. Notably, pPlGF1 gene transfer not only increased the number of mononuclear cells in the bone marrow (BM-MNCs) in BMT/MI/GTx (P<0.01 for pPlGF1 vs Mock and PBS) **(Figure S10*A*)**, but also increased the fraction of GFP^+^Sca-1^+^lin^–^ (GSL) cells in this population of BM-MNCs at day 7 and 28 (P<0.01) **(Figure S10*B*,*C*)**. Thus, pPlGF1 transfer stimulated the expansion of the BM progenitor cell pool *in vivo*.

To further investigate the mitogenic mechanism of pPlGF1 on BM-SL cells, we assessed the PI3-kinase/Akt and MAPK signaling pathway. Immunoblotting revealed that pPlGF1 transfer increased the levels of phospho-Akt and of the phospho-p42/p44 mitogen-activated protein kinases (known to be involved in cell growth [3]) in BM-SL cells, isolated from BMT/MI/GTx on day 7 **(Figure S10*D*)**. Thus, PlGF gene transfer activated the PI3-kinase and MAPK signaling pathway in BM-SL cells and stimulated their expansion.

**pPlGF1 gene transfer activates cell growth through induction of Akt and MAPK pathways *in vivo*.**

We performed double immunohistochemistry for phospho-Akt and isolectin B4 to identify double positive cells as growing endothelial cells in the infarcted myocardium **(Figure S11*A,B*)**. Double staining for activated MAPK and isolectin B4 **(Figure S11*C,D*)** and that for activated MAPK and cTnI **(Figure S12*A,B*)** were also performed to detect growing endothelial cells and cardiomyocytes, respectively. Seven days after gene transfer, the number of growing endothelial cells was significantly greater in the pPlGF1 group than in the Mock and PBS groups. In contrast, the number of growing cardiomyocytes was similar in all groups **(Figure S11,12)**. These results suggest that PlGF gene transfer may enhance the growth of cardiac endothelial cells but not cardiomyocytes through Akt and MAPK pathways.

**pPlGF gene transfer stimulates vascular regeneration**

To expand on our observation that pPlGF1 gene transfer is capable of stimulating the differentiation of BM-SL cells into endothelial cells, we isolated SL cells from the BM of BMT/MI/GTx rats and used a previously established EPC colony formation assay (EPC-CFA) to count the small and large colony forming units (CFUs), containing more immature or mature EPCs, respectively [4]. Compared to controls, pPlGF1 gene transfer stimulated the vasculogenic potential of BM-SL cells. Indeed, the total number of EPC-CFUs was 12.5 ± 1.6 after pPlGF1 and only 4.0 ± 0.9 after Mock and 5.3 ± 1.3 after PBS (P<0.05 for pPlGF1 vs Mock and PBS) **(Figure S13)**. Subtyping of EPC-CFUs revealed that pPlGF1 gene transfer stimulated the differentiation of both small and large EPC colonies **(Figure S13).** Thus, pPlGF1 transfer stimulated the vasculogenic commitment and differentiation of the BM-SL cells, which likely contributed to the increased myocardial revascularization.

***References***

1. Maglione D, Guerriero V, Viglietto G, Ferraro MG, Aprelikova O, et al . (1993) *Oncogene* 8, 925-31.

2. Green CJ, Lichtlen P, Huynh NT, Yanovsky M, Laderoute KR, et al. (2001) *Cancer Res* 61, 2696-703.

3. Chen X, Mao Z, Liu S, Liu H, Wang X, et al. (2005) Dedifferentiation of adult human myoblasts induced by ciliary neurotrophic factor in vitro. *Mol Biol Cell* 16:3140-51.

4. Masuda H, Yoshida M, Kobori M, Itoh R, Sadamoto K, et al. (2006) *Circ J*, 361. Abstract.
